# Enoyl-CoA hydratase-1 regulates mTOR signaling and apoptosis by sensing nutrients

**DOI:** 10.1038/s41467-017-00489-5

**Published:** 2017-09-06

**Authors:** Ya-Kun Zhang, Yuan-Yuan Qu, Yan Lin, Xiao-Hui Wu, Hou-Zao Chen, Xu Wang, Kai-Qiang Zhou, Yun Wei, Fushen Guo, Cui-Fang Yao, Xia-Di He, Li-Xia Liu, Chen Yang, Zong-Yuan Guan, Shi-Dong Wang, Jianyuan Zhao, De-Pei Liu, Shi-Min Zhao, Wei Xu

**Affiliations:** 1 Obstetrics & Gynecology Hospital of Fudan University, State Key Lab of Genetic Engineering, Institutes of Biomedical Sciences and School of Life Sciences, Shanghai, 200011 China; 2 Key Laboratory of Reproduction Regulation of NPFPC, Collaborative Innovation Center for Genetics and Development, Shanghai, 200433 China; 3 State Key Laboratory of Biotherapy/Collaborative Innovation Center for Biotherapy, West China Hospital, Sichuan University, Chengdu, 610041 China; 4 Department of Urology, Fudan University Shanghai Cancer Center, Shanghai, 200032 China; 5 Department of Oncology, Shanghai Medical College, Shanghai, 200032 China; 6 Institute of Developmental Biology and Molecular Medicine, Fudan University, Shanghai, 200032 China; 7 State Key Laboratory of Medical Molecular Biology, Institute of Basic Medical Sciences, Chinese Academy of Medical Sciences & Peking Union Medical College, Beijing, 100010 China; 8 Institute of Plant Physiology and Ecology, Shanghai Institutes for Biological Sciences, Chinese Academy of Sciences, Shanghai, 200031 China; 9 Sophie Davis School of Biomedical Education, City University of New York Medical School, New York, NY 10031 USA; 10 Vanderbilt University School of Medicine, Nashville, TN 37232 USA

## Abstract

The oncogenic mechanisms of overnutrition, a confirmed independent cancer risk factor, remain poorly understood. Herein, we report that enoyl-CoA hydratase-1 (ECHS1), the enzyme involved in the oxidation of fatty acids (FAs) and branched-chain amino acids (BCAAs), senses nutrients and promotes mTOR activation and apoptotic resistance. Nutrients-promoted acetylation of lys^101^ of ECHS1 impedes ECHS1 activity by impairing enoyl-CoA binding, promoting ECHS1 degradation and blocking its mitochondrial translocation through inducing ubiquitination. As a result, nutrients induce the accumulation of BCAAs and FAs that activate mTOR signaling and stimulate apoptosis, respectively. The latter was overcome by selection of BCL-2 overexpressing cells under overnutrition conditions. The oncogenic effects of nutrients were reversed by SIRT3, which deacetylates lys^101^ acetylation. Severely decreased ECHS1, accumulation of BCAAs and FAs, activation of mTOR and overexpression of BCL-2 were observed in cancer tissues from metabolic organs. Our results identified ECHS1, a nutrients-sensing protein that transforms nutrient signals into oncogenic signals.

## Abstract (web summary)

Overnutrition has been linked to increased risk of cancer. Here, the authors show that exceeding nutrients suppress Enoyl-CoA hydratase-1 (ECHS1) activity by inducing its acetylation resulting in accumulation of fatty acids and branched-chain amino acids and oncogenic mTOR activation.

## Introduction

Overnutrition has become a major health care challenge given that it increases the risks for a number of major human diseases. For example, epidemiological studies indicate that overnutrition is linked to increased risks of various cancers^1^. However, the mechanisms involved in the promotion of cancer onset by nutrients remain poorly understood. The increase in insulin induced by nutrients is hypothesized to be a cause of cancers, given that human cancers produce/secrete insulin or depend on insulin^2^. Moreover, activation of the mammalian target of rapamycin (mTOR) signaling^3^ by nutrients, the master regulator of anabolic metabolism, is also proposed as a cause of overnutrition-associated cancers because mTOR signaling is commonly activated in cancer cells^4^. However, the activation of mTOR signaling in cancer cells is often due to mutations in oncogenes, such as in PI3K, Ras, and Raf, growth factor receptor kinases, autocrine growth factors^5–7^, and tumor suppressors such as PTEN, AMPK, TSC2, LKB1, and NF1^8, 9^. There is insufficient direct evidence that overnutrition is directly associated with an increase of such mutations. Moreover, nutrient-promoted insulin secretion and mTOR activation are temporary and reversible processes that are relieved by nutrient removal, indicating that overnutrition does not promote cancer onset through promoting mutations. Creation of environments that facilitate the survival of cells with cancerous mutations is another possible mechanism for nutrients to induce cancer^10^. However, overnutrition such as high glucose promotes apoptosis in endothelial cells, β-cells and in human hepatoma HepG2 cells^11, 12^, showing that alternative mechanisms underlying the tumorigenic effects of overnutrition.

To support continuous growth, one of the signatures of cancer cells, new cellular components including proteins, nucleic acids, and lipids need to be accumulated. Fatty acids (FAs), the building blocks of lipid biosynthesis, is accumulated in cancer cells through either increasing of de novo biosynthesis^13^ and/or decreasing oxidation of FAs^14, 15^. Protein synthesis in cancer cells is sustained, at least partly, by the constitutive activation of mTOR signaling. Notably, elevated circulating branched-chain amino acids (BCAAs), the potent physiological activators of mTOR signaling and protein synthesis^16^, but not other amino acids, were identified as an early event in pancreatic cancer progression^17^. Based on this evidence, FAs and BCAAs need to be co-accumulated in cancer cells.

Existing evidence support the notion that metabolism of FAs and BCAAs are co-regulated. Extensive exercise promoted the simultaneous oxidation of FAs and BCAAs in mice^18^. Remarkably, the accumulation of both FAs and BCAAs is often observed in overnutrition-associated insulin resistance, diabetes, and obesity. Moreover, although BCAAs are synthesized from distinct pathways, they are co-oxidized through β-oxidation^19^. Furthermore, overnutrition-associated diseases are all associated with higher cancer risks^20–22^, indicating that metabolism of BCAAs and FAs may be related to cancer onset.

Enoyl-CoA hydratase 1 (ECHS1; EC 4.2.1.17), the enzyme that catalyzes the hydration of medium- and short-chained enoyl-CoAs^23^, is the converging enzyme of oxidation of both FAs and BCAAs. ECHS1 protein levels are downregulated by a high fat diet in Sprague–Dawley rats^24^ and by high glucose levels in a C57BL/6J diabetes mouse model^25^, suggesting that ECHS1 senses nutrient signals. Therefore, whether and how ECHS1 regulates FAs and BCAAs catabolism and cellular FAs and BCAAs levels is of importance in efforts to understand the tumorigenic impact of overnutrition.

We and others previously found that protein lysine acetylation (referred to as acetylation hereafter) is a general nutrient sensing mechanism that accesses the availability of acetyl-CoA, the universal signal of nutrient abundance, in cells^26, 27^. Acetylation senses nutrients and coordinates cellular metabolism such as fatty acid oxidation^26, 28^ and amino acid metabolism associated metabolic enzymes^29, 30^, via distinct mechanisms, including activation^26^, inactivation^28, 31^, and destabilization^32, 33^. In the current study, we investigated whether, and just how nutrients coordinate FAs and BCAAs oxidation through ECHS1 acetylation, and how this regulation promotes the initiation of cancer.

## Results

### Regulation of ECHS1 protein levels by nutrients

**Fig. 1:**
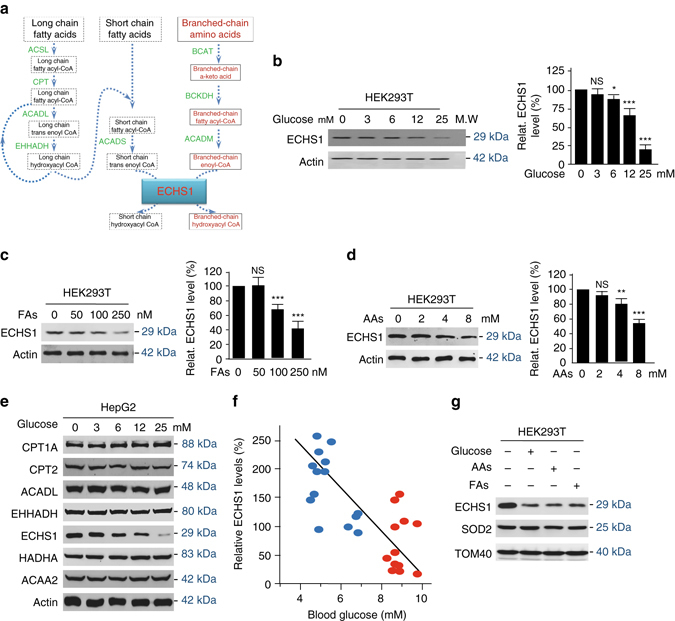
ECHS1 protein levels are decreased by nutrients. **a** Schematic diagram of catabolic pathways of FA (*black-colored*) and BCAA (*red-colored*) oxidation that involve ECHS1. Long chain FAs are oxidized to short-chained intermediates prior to being completely oxidized through ECHS1. BCAAs are oxidized through ECHS1. ACADL acyl-CoA dehydrogenase, long chain, ACADM medium-chain specific acyl-CoA dehydrogenase, mitochondrial, ACADS short-chain specific acyl-CoA dehydrogenase, ACSL acyl-CoA synthetase long-chain, BCAT branched-chain amino-acid transaminase, BCKDH branched-chain keto acid dehydrogenase, carnitine palmitoyltransferase, ECHS1 enoyl CoA hydratase, short chain 1, mitochondrial, EHHADH Enoyl-CoA, Hydratase/3-Hydroxyacyl CoA Dehydrogenase. **b**–**d** Endogenous ECHS1 levels were detected in HEK293T cells treated with different concentrations of glucose (**b**), FAs (linoleic acid + palmitic acid) (**c**), and amino acids (glutamate + aspartate) (**d**). Representative western blot results and quantitation (herein after) of triplicated western blot are shown. **e** HepG2 cells were exposed to indicated concentrations of glucose. Levels of endogenous β-oxidation enzymes were detected 4 h after glucose exposure. CPT1A Carnitine O-palmitoyltransferase 1, liver isoform, Carnitine O-palmitoyltransferase 2, mitochondrial, HADHA Trifunctional enzyme subunit alpha, mitochondrial, ACAA2 3-ketoacyl-CoA thiolase, mitochondrial. **f** Plasma ECHS1 levels of normal people (*blue*, *n* = 14) and untreated patients with diabetes (*red*, *n* = 12) were determined by western blots. Relative ECHS1 levels (normalized to the average ECHS1 level) were plotted against serum glucose levels. **g** Mitochondria of HEK293T cells cultured in DMEM base, DMEM base supplemented with glucose (25 mM), fatty acids (250 nM) and amino acids (8 mM), respectively, were isolated and the relative (to SOD2 and TOM40) mitochondrial ECHS1 levels were compared. For **b**–**d**, mean values of quantitation with SD are reported. *P < 0.05; **P < 0.01; ***P < 0.001

ECHS1 is at the converging point of the oxidation of FAs and BCAAs (Fig. 1a), suggesting that ECHS1 may simultaneously control the catabolism of FAs and BCAAs. In mice, Echs1 protein levels are decreased by nutrients^24, 25^, suggesting a possibility that ECHS1 senses nutrients. We confirmed this hypothesis in different systems. In cultured cells, endogenous ECHS1 protein levels of human embryonic kidney HEK293T cells, 786-O and ACHN renal cell cancer cells, SMMC7712 and Huh7 liver cancer cells, and HCT 116 and SW620 colon cancer cells were severely decreased, while ECHS1 protein levels of LNCaP and PC3 prostate cancers cells were moderately downregulated by exposure to glucose, FAs, and amino acids, respectively (Fig. 1b–d, Supplementary Fig. 1). This demonstrated that nutrients differentially downregulate ECHS1 levels in particular in cell lines derived from metabolic organs. Moreover, exposure to glucose dose-dependently decreased endogenous ECHS1 levels, but not those of enzymes functioning either upstream or downstream of ECHS1 in human liver carcinoma HepG2 cells (Fig. 1e). These results showed that ECHS1 senses nutrients in cultured cells. Furthermore, the circulating ECHS1 protein levels of untreated type 2 diabetes patients and healthy individuals who were between 40 and 68 years of age and with fasting blood glucose levels between 4 and 10 mM, were inversely correlated to their blood glucose levels (Fig. 1f, Supplementary Fig. 2), confirming that ECHS1 levels are controlled by nutrient levels in vivo. Lastly, we found mitochondrial ECHS1 protein levels reflect the functionality of ECHS1 due to the fact that ECHS1 localized to mitochondria were negatively regulated by glucose, fatty acids, and amino acids (Fig. 1g), assuring the physiologic importance of ECHS1 protein level regulation by nutrients.

### Nutrients promote ECHS1 acetylation on Lys^101^

**Fig. 2:**
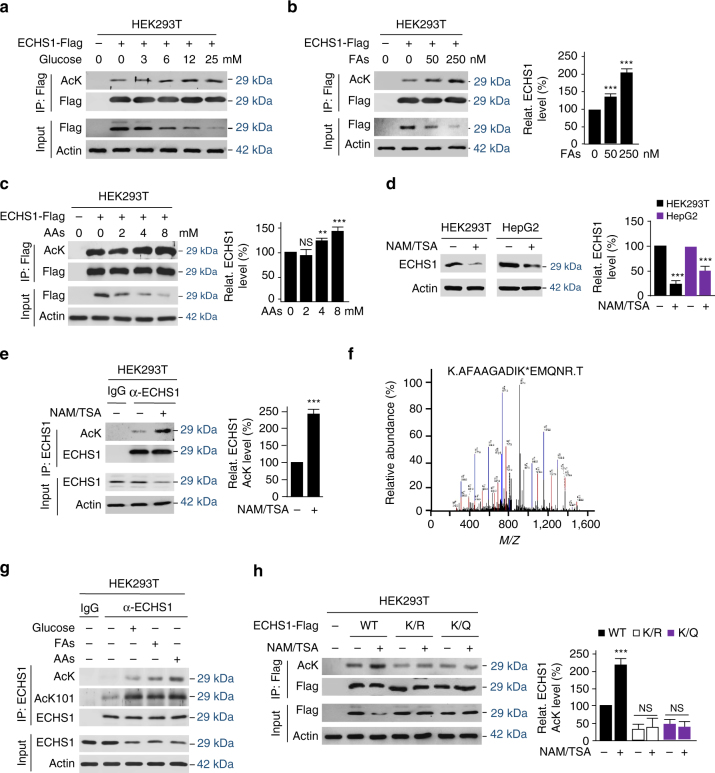
Nutrients promote ECHS1 K101 acetylation. **a**–**c** Western blot detection of acetylation levels of ectopically expressed ECHS1 in HEK293T cell cultured in the presence of excessive glucose (**a**), FAs (**b**), and amino acids (**c**) in the culture media. **d** Levels of endogenous ECHS1 in HEK293T and HepG2 cells were compared with and without the presence of deacetylases inhibitors NAM/TSA. **e** Effects of deacetylase inhibitors on the acetylation levels of affinity-purified endogenous ECHS1. **f** The MS spectrum that led to the identification of K101 acetylation containing tryptic peptide from endogenous ECHS1. **g** Western blot detection of proteins levels, AcK levels and AcK101 levels of endogenous ECHS1 in HEK293T cells that were cultured in DMEM base and DMEM base supplemented with glucose (25 mM), fatty acids (250 nM), and amino acids (8 mM). **h** Flag-tagged wildtype, K101R (K/R), and K101Q (K/Q) ECHS1 were overexpressed in HEK293T cells that were cultured in media with or without NAM/TSA. The relative acetylation levels of ECHS1 proteins purified from the above mentioned cells using Flag beads was determined. For **b**–**e** and **h**, mean values of quantitation with SD are reported. *P < 0.05; **P < 0.001; ***P < 0.001

Acetylation is known to sense nutrients^26^. To test whether ECHS1 senses nutrients via acetylation, we first confirmed that the acetylation levels of ectopically expressed ECHS1 from HEK293T and other cultured cancer cells, probed by a pan-acetyl lysine antibody (α-AcK)^26^, were each increased by glucose, FAs, and amino acids (Fig. 2a–c, Supplementary Fig. 3). Moreover, the endogenous ECHS1 levels in both HEK293T cells and HepG2 cells were downregulated by treatment with the deacetylases inhibitors, nicotinamide (NAM), and trichostatin A (TSA) (Fig. 2d), simulating the effects of nutrient exposure. These results confirmed that ECHS1 is regulated through acetylation, a notion further supported by evidence that affinity-purified endogenous ECHS1 from NAM/TSA treated HEK293T cells was more acetylated than untreated HEK293T cells (Fig. 2e).

Employing α-AcK enrichment of acetylated tryptic peptides from hyper-acetylated endogenous ECHS1 followed by mass spectrometry sequencing, we confirmed that lysine 101 (K101) in ECHS1 was acetylated (Fig. 2f). Moreover, employing pan-acetyl-lysine and a homemade anti-acetyl-lysine101 antibody (α-AcK101) (Supplementary Fig. 4), we found that both the AcK and AcK101 levels were increased by nutrients (Fig. 2g). Furthermore, the deacetylase inhibitors NAM/TSA mix treatments only increased the acetylation level of wild-type ECHS1, but not that of the acetylation mimetic K101Q (K/Q) or non-acetylable K101R (K/R) mutants, both presenting diminished acetylation signals (Fig. 2h), suggested that K101 is the major acetylation site in ECHS1.

### GCN5 acetylates K101 in ECHS1

**Fig. 3:**
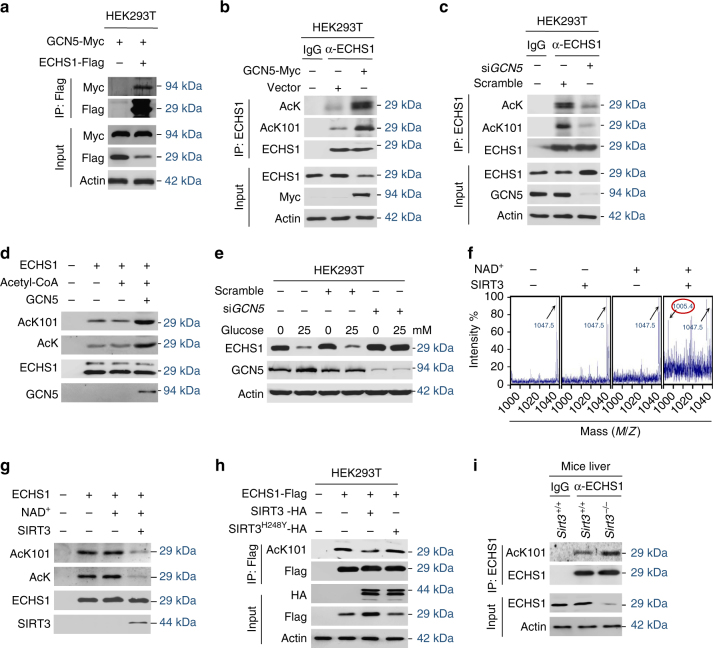
GCN5 acetylates and SIRT3 deacetylates ECHS1. **a** Myc-tagged GCN5 and Flag-tagged ECHS1 were co-expressed in HEK293T cells. The co-purified Myc-tagged GCN5 was detected in Flag beads purified proteins. **b**, **c** Western blot detection of endogenous ECHS1, and AcK and AcK101 levels of affinity-purified ECHS1 from HEK293T cells and GCN5 overexpressing HEK293T cells (**b**) and from HEK293T cells and *GCN5* knockdown HEK293T cells (**c**). **d** Western blot detection of AcK and AcK101 levels of recombinant ECHS1 that before and after in vitro acetylation by GCN5. **e** The levels of endogenous ECHS1 were detected in HEK293T and *GCN5* knockdown HEK293T cells that were cultured in the absence of glucose or presence of glucose. **f** The synthetic acetylated K101-containing ECHS1 peptide was deacetylated by recombinant SIRT3. The synthetic peptide (1047.5) and de-acetylated products (1005.4, *red-circled*) were assayed by MS. **g** The levels of AcK and AcK101 of hyperacetylated ECHS1 (from NAM treated cells) were detected before and after in vitro treatment by SIRT3. **h** ECHS1 was co-expressed with SIRT3 or SIRT3^H248Y^ in HEK293T cells. The AcK101 levels of purified ECHS1 were determined. **i** The Ac101 levels of affinity-purified ECHS1 from liver of *Sirt3* KO and control mice

Screening for the acetyltransferase involved in the acetylation of ECHS1 revealed that general control of amino acid synthesis 5 (GCN5)^34^, but not CREB binding protein (CBP)^35^, E1A binding protein p300 (P300)^36^, or acetyl-CoA acetyltransferase (ACAT1)^37^, increased AcK101 and decreased protein levels of ECHS1 (Supplementary Fig. 5a). This was consistent with the fact that GCN5 interacted with ECHS1 when they were co-expressed in HEK293T cells (Fig. 3a), and that *GCN5* overexpression increased, but *GCN5* knockdown decreased AcK and AcK101 levels of endogenous ECHS1 (Fig. 3b, c) and ectopically expressed ECHS1 in HEK293T cells (Supplementary Fig. 5b). Moreover, affinity-purified GCN5 acetylated ECHS1 K101 in vitro (Fig. 3d), confirmed that GCN5 is an acetyltransferase of ECHS1. Furthermore, *GCN5* knockdown not only increased endogenous ECHS1 protein levels, but also abrogated glucose-promoted ECHS1 decrease in HEK293T cells (Fig. 3e), suggesting that GCN5 is the major acetyltransferase for ECHS1. Lastly, GCN5 overexpression increased AcK101 levels of ECHS1 wildtype, but not that of the K101R mutant (Supplementary Fig. 5c), confirming that GCN5 acetylates K101 in ECHS1.

### SIRT3 deacetylates K101 in ECHS1

Since only the type III deacetylase inhibitor NAM^38^, but not type I, II, and IV deacetylase inhibitor TSA^39^, increased K101 acetylation in ECHS1 (Supplementary Fig. 5d), sirtuins were screened for their ability to deacetylate ECHS1. SIRT3, a mitochondrial- and cytosol-dual-functioning type III deacetylase^40^, was identified as a candidate deacetylase for ECHS1 because it interacted with ECHS1, while the other tested sirtuins did not (Supplementary Fig. 5e, f). Moreover, SIRT3 deacetylated synthetic AcK101-containing ECHS1 peptide in an NAD^+^-dependent manner in vitro (Fig. 3f), and recombinant SIRT3 decreased AcK and AcK101 levels in affinity purified ECHS1 in vitro (Fig. 3g), confirmed its role as ECHS1 deacetylase. Furthermore, co-expression of SIRT3, but not that of a catalytic-dead^41^ SIRT3^H248Y^, SIRT4, or SIRT5, with ECHS1 decreased AcK101 levels (Fig. 3h, Supplementary Fig. 5g), and *Sirt3* knockout in 129/C57BL6 mice (*Sirt3* ^*−/−*^ mice) increased AcK101 levels of endogenous ECHS1 in the liver of mice (Fig. 3i), indicating that SIRT3 deacetylates ECHS1 both in vitro and in vivo.

Notably, the expression of SIRT3, but not GCN5, was decreased by glucose, fatty acids, and amino acids (Supplementary Fig. 5h), consistent with SIRT3 expression being downregulated by nutrients^42^, and suggests that fatty acids and amino acids decrease ECHS1 levels through SIRT3 downregulation-induced ECHS1 hyperacetylation.

### K101 acetylation impairs ECHS1 catalytic activity

**Fig. 4:**
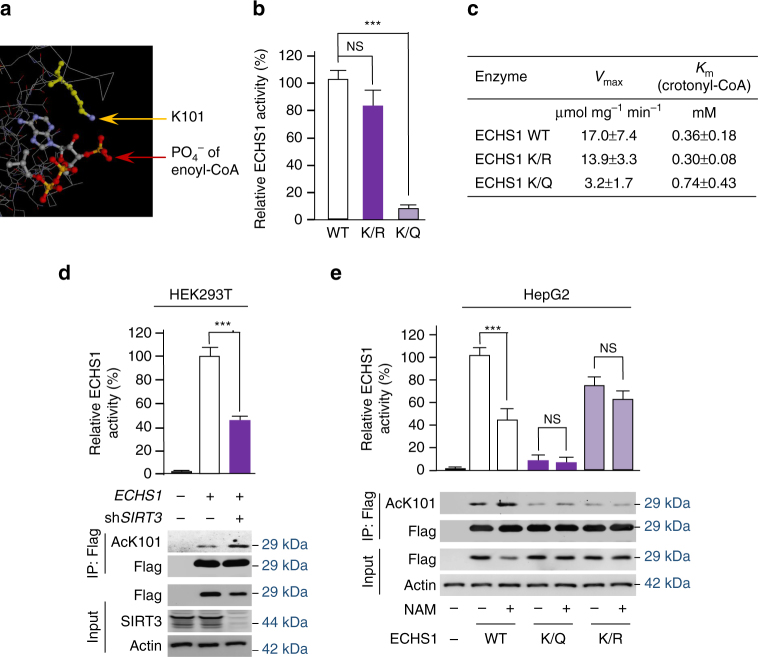
Acetylation inactivates ECHS1. **a** The crystal structure shows that K101 is involved in enoyl-CoA binding. Arrows point to the amine on lysine (*yellow*) and the phosphate on enoyl-CoA (*red*). **b** The relative specific activities of ECHS1 wild-type (set as 100%), K/R, and K/Q mutants were determined. Mean values with SD are reported. *NS* not significant; ***P < 0.001. **c** The *V*_max_ and *K*_m_ were determined for wild type ECHS1, K/R, and K/Q mutant using crotonyl-CoA as the substrate. **d** The activities of ECHS1 expressed in HEK293T or *SIRT3* knocked down HEK293T cells were determined. **e** The K101 acetylation levels and relative specific activities of Flag-tagged wild type, K/Q, and K/R mutant ECHS1 expressed in HepG2 cells cultured in the presence or absence of NAM (5 mM). The average specific activities (*n* = 3) with SD and representative western blots are shown

The positively charged ε-amine of K101 in ECHS1 is involved in the binding of the negatively charged phosphate group of enoyl-CoA (Fig. 4a)^43^, ECHS1 substrate, suggesting that K101 acetylation would impede enoyl-CoA binding to ECHS1. Consistent with this result, replacing the lysine by a glutamine (K/Q) to remove K101 positive charge virtually abolished ECHS1 catalytic activity, while replacing the lysine by an arginine (K/R) to preserve the positive charge barely affected ECHS1 catalytic activity (Fig. 4b). Kinetic studies using ECHS1 and its mutants revealed that the *K*_m_ of the K/Q mutant for enoyl-CoA doubled, while the *V*_max_ decreased by about 5-fold when compared to the *K*_m_ and *V*_max_ of the wildtype ECHS1, respectively. However, the K/R mutant presented kinetic parameters similar to those of the wild-type ECHS1 (Fig. 4c). Moreover, the specific activity of ECHS1 ectopically expressed in *SIRT3* knockdown HEK293T cells was 50% less than that from ECHS1 produced in HEK293T cells (Fig. 4d). Additionally, the specific activity of wildtype ECHS1, but not that of K/Q or K/R mutants, was inhibited by NAM treatment when overexpressed in HepG2 cells (Fig. 4e). These results confirmed that K101 acetylation inactivates ECHS1.

### SH3RF2 mediates ubiquitination of K101-acetylated ECHS1

**Fig. 5:**
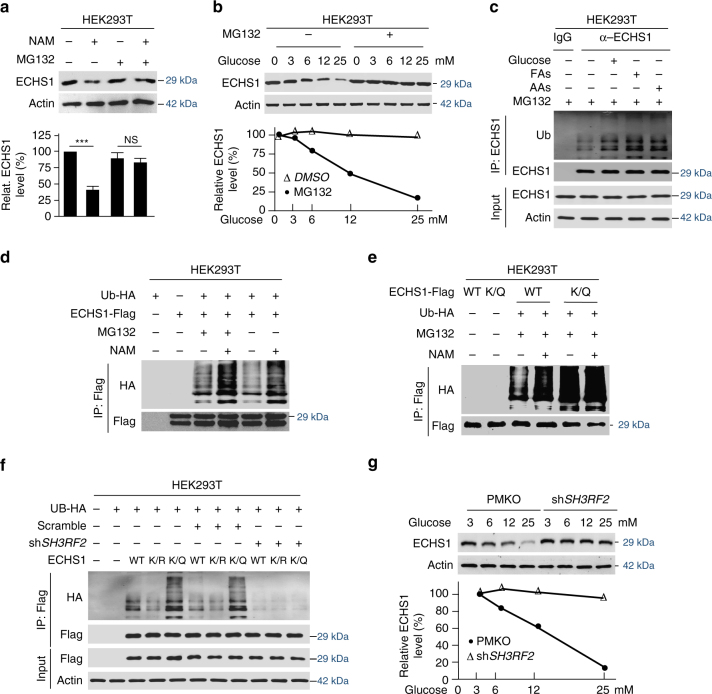
K101 acetylation promotes ECHS1 ubiquitination and degradation. **a** NAM treatment effects on endogenous ECHS1 levels in HEK293T cells were analyzed in the presence and absence of MG132. Mean values of quantitation with SD are reported. NS not significant; ***P < 0.001. **b** The endogenous ECHS1 levels in HEK293T cells that were cultured under various glucose levels were detected under absence (DMSO) and presence treatments with MG132. **c** The ubiquitination of affinity-purified endogenous ECHS1 from HEK293T cells that were cultured with DMEM base and DMEM base supplemented with glucose, fatty acids, and amino acids, respectively, were determined. **d** Flag-tagged ECHS1 was expressed in HEK293T cells. The ubiquitination levels of ECHS1 expressed from cells cultured with either NAM or MG132, or both NAM and MG132 treatments were analyzed. **e** Flag-tagged ECHS1 or K/Q mutant were expressed alone or with HA-tagged ubiquitin in HEK293T cells cultured with or without NAM. The ubiquitination levels of Flag bead affinity-purified ECHS1 were analyzed. **f** The ubiquitination levels of ectopically expressed Flag-tagged ECHS1, K/R and K/Q mutants from HEK293T and sh*SH3RF2* knockdown (KD effects please see Supplementary Fig. 7d) HEK293T cells. **g** Endogenous ECHS1 levels were detected in HEK293T cells and *SH3RF2* knocked down (KD effects please see Supplementary Fig. 7d) HEK293T cells that were cultured in media contained indicated glucose levels. Relative ECHS1 levels were all normalized to that of glucose of 3 mM

In an attempt to elucidate the mechanism underlying the nutrient-induced decrease in ECHS1 protein levels, we found that glucose exposure resulted in a decrease in endogenous ECHS1 protein levels (Fig. 1b, e). However, this was not due to altered *ECHS1* mRNA transcription (Supplementary Fig. 6a). Moreover, blockade of protein translation by cycloheximide^44^ failed to prevent glucose-induced ECHS1 decrease (Supplementary Fig. 6b), and the proteasomal inhibitor MG132 blocked the NAM- (Fig. 5a) and high glucose-induced (Fig. 5b) decrease of endogenous ECHS1 protein levels. Furthermore, nutrients promoted ubiquitination of ECHS1 in HEK293T cells (Fig. 5c). These results suggest that nutrient-enhanced acetylation promotes proteasomal degradation of ECHS1, which was further confirmed by the fact that MG132 and NAM treatments synergistically enhanced ECHS1 ubiquitination (Fig. 5d). Moreover, the ubiquitination level of the acetylation mimetic K/Q mutant, which was more highly ubiquitinated than wildtype ECHS1, was not further enhanced by NAM treatment (Fig. 5e). These results collectively showed that K101 acetylation promotes ECHS1 ubiquitination.

Further confirming that ECHS1 is ubiquitinated, we identified the SH3-domain-containing RING finger protein (SH3RF2), a known member of the cytosolic SH3RF E3 ligase family^45–47^, as the candidate E3 ligase for ECHS1 by employing tandem affinity purification (Supplementary Fig. 7a). SH3RF2 interacted with ECHS1 (Supplementary Fig. 7b) and SH3RF2 overexpression decreased endogenous ECHS1 levels (Supplementary Fig. 7c). Conversely, SH3RF2 depletion by *shRNA* in HEK293T cells resulted in a decrease in overexpressed (Supplementary Fig. 7d) and endogenous ECHS1 ubiquitination (Supplementary Fig. 7e) and accumulation of ECHS1 in cells (Supplementary Fig. 7f). These results confirmed that SH3RF2 is the E3 ligase for ECHS1. Moreover, when one depletes SH3RF2 from HEK293T cells, it abrogates ubiquitination of wildtype, non-acetylable K/R mutant, and the acetylation mimetic K/Q mutant, the latter two had lower ubiquitination and had higher ubiquitination than that of wildtype ECHS1, respectively (Fig. 5f). Furthermore, *SH3RF2* knockdown abrogated the glucose-induced ECHS1 decrease in HEK293T cells (Fig. 5g). These results collectively confirmed that glucose regulates ECHS1 through K101 acetylation promoted SH3RF2-mediated ECHS1 ubiquitination.

### Acetylation blocks ECHS1 mitochondria translocation

**Fig. 6:**
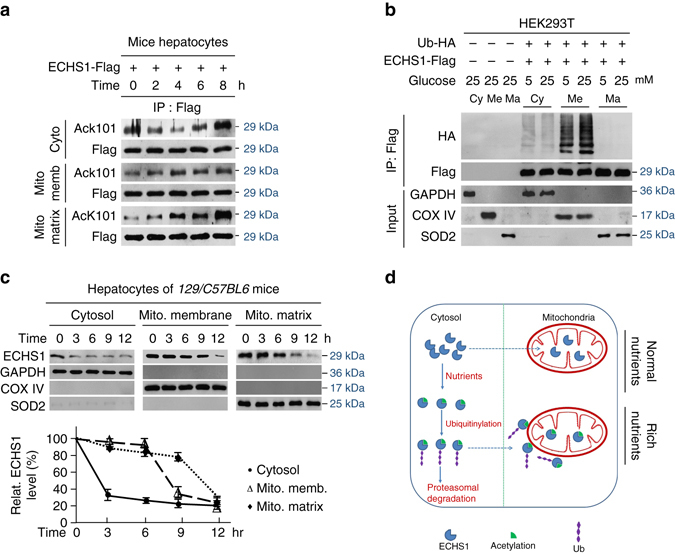
K101 acetylation inhibits ECHS1 mitochondria translocation. **a** The K101 acetylation levels of ECHS1 in the cytosol, mitochondria membrane, and mitochondria matrix were detected after mouse primary hepatocytes were exposed to 25 mM glucose for 0, 2, 4, 6, and 8 h. **b** Flag-tagged ECHS1 was co-expressed with HA-tagged ubiquitin in HEK293T cells. ECHS1 ubiquitination levels in the cytosol, mitochondria membrane, and mitochondria matrix were detected under low and high glucose treatment for 4 h. GAPDH, COX IV and SOD2 were used as markers of cytosol, mitochondria membrane and mitochondria matrix, respectively. **c** ECHS1 levels in the cytosol, mitochondria membrane, and mitochondria matrix were analyzed (*n* = 3) after cells were exposed to 25 mM glucose for 3, 6, 9, and 12 h. Representative western blots are presented. All levels were normalized to those of cells at time 0 and the average relative levels were quantified (*bottom*). **d** Schematic diagram of ECHS1 regulation by nutrients. Nutrient-induced acetylation inactivates ECHS1 and induces ubiquitination of ECHS1 that blocks ECHS1 mitochondrial translocation and promotes ECHS1 degradation. These effects synergistically inactivate ECHS1

To determine how cytosolic-occurring ubiquitination regulates mitochondrial ECHS1, we traced ECHS1 acetylation and ubiquitination levels in different cellular compartments. Upon glucose exposure, ECHS1 AcK101 levels was drastically increased in the mitochondria matrix and moderately increased in the cytoplasm and mitochondria membranes in a time-dependent manner (Fig. 6a), showing that acetylation did not affect ECHS1 translocation to the mitochondria. However, ubiquitinated ECHS1 was detected in the cytosol and in the mitochondria membrane, but not in the mitochondria matrix. Moreover, ubiquitinated ECHS1 was enriched in the mitochondria membrane and glucose exposure exaggerated the enrichment (Fig. 6b). These results, consistent with observations that glucose exposure induced a much quicker ECHS1 decrease in the cytosol than in either the mitochondrial membrane and matrix in primary hepatocytes of mice (Fig. 6c), demonstrated that ubiquitinated ECHS1 lost its ability to penetrate the mitochondrial membrane. Taken together, nutrient-induced acetylation inactivates ECHS1 through synergistically impairing its catalytic activity and inducing its ubiquitination, thereby promoting its cytosolic degradation and preventing its translocation to the mitochondria (Fig. 6d).

### ECHS1 inactivation activates mTOR signaling

**Fig. 7:**
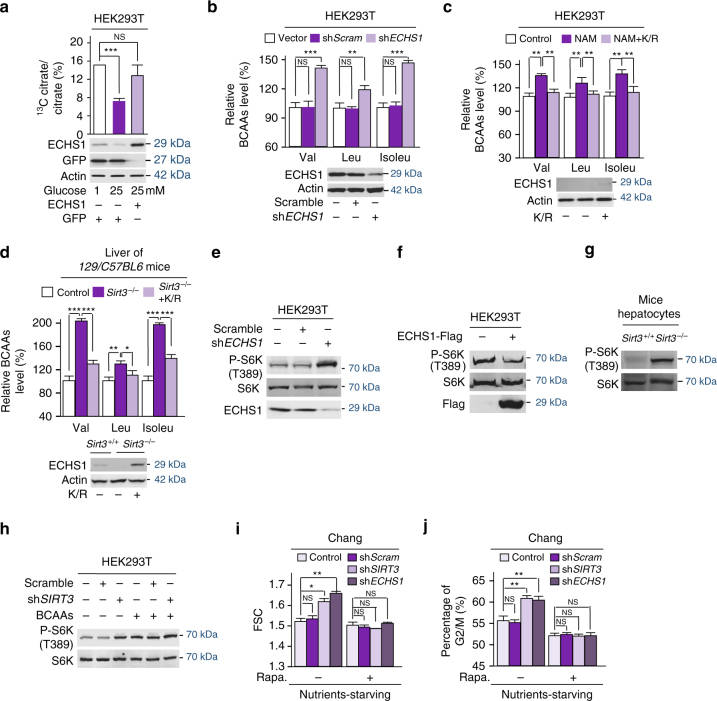
Nutrient activates mTOR signaling by accumulating BCAAs. **a** The conversion of Leucine-1,2-^13^C_2_ to ^13^C-labeled citrate was traced by targeted liquid-chromatography tandem mass spectrometry (LC–MS/MS) in HEK293T cells cultured in low (1 mM) and high (25 mM) glucose media, and in ECHS1 overexpressing HEK293T cells cultured in high glucose media. **b** The BCAA levels in HEK293T cells and ECHS1 knocked down HEK293T cells were determined (*n* = 4). BCAA levels were normalized to those of HEK293T cells. **c** BCAA levels of HEK293T cells, NAM treated HEK293T cells, and NAM treated HEK293T cells with overexpression of ECHS1^K101R^ were determined by GC–MS (*n* = 4). All levels were normalized to those of HEK293T cells. The expression of ECHS1^K/R^ was confirmed by western blot. **d** BCAAs levels in the liver of 129/C57BL6 mice, *Sirt3* knockout 129/C57BL6 mice, and ECHS1^K101R^ overexpressing *Sirt3* knockout 129/C57BL6 mice were determined (*n* = 6). ECHS1^K/R^ expression was achieved by tail vein injection of the ECHS1^K/R^ plasmid, and the expression was confirmed by western blot. BCAA levels were normalized to those in the liver of isogenic control mice. **e** The levels of P-T389 on S6K were determined in HEK293T cells with and without *ECHS1* knockdown. **f** The levels of P-T389 on S6K were determined in HEK293T cells with and without ECHS1 overexpression. **g** The levels of P-T389 on S6K were determined in the hepatocytes of *Sirt3* KO and its isogenic control mice. **h** The effects of sh*SIRT3* knockdown (for knockdown effects see Figure 4) on P-T389 of S6K were detected in HEK293T cells cultured with or without BCAA supplementation in the medium. **i** The cell size of Chang cells and *ECHS1* or *SIRT3* knocked down Chang cells were determined by measuring forward side scatter (FSC) in the presence or absence of rapamycin. **j** The percentage of Chang cells and *ECHS1* or *SIRT3* knocked down Chang cells in the G2/M phase was determined by fluorescence-activated cell sorter (FACS) in the presence or absence of rapamycin. For all figures, mean values with SD are reported. NS not significant; *P < 0.05; **P < 0.01; ***P < 0.001

In HEK293T cells, the utilization of leucine, traced by conversion of Leucine-1,2-^13^C_2_ to ^13^C-labeled citrate^48^, is suppressed by high glucose exposure in an ECHS1 overexpression reversible manner (Fig. 7a), this was similar to the *ECHS1* knockdown in the same cell line leading to 39%, 20%, and 44% increases in the levels of valine, leucine, and isoleucine (Fig. 7b), respectively, supported that glucose-regulated ECHS1 controls BCAA oxidation. The lower degree of leucine accumulation by *ECHS1* knockdown may be explained by the fact that methylglutaconyl-CoA hydratase, another hydratase, functions alongside with ECHS1 and oxidizes leucine^49^. Moreover, confirming that SIRT3 deacetylates and activates ECHS1, NAM treatment (Fig. 7c), *SIRT3* knockdown (Supplementary Fig. 8a) in HEK293T cells, and *Sirt3* knockout (*Sirt3* ^*−/−*^) in liver of *129/C57BL6* mice (Fig. 7d) increased cellular BCAA levels in a non-acetylable active ECHS1^K101R^ mutant overexpression reversible manner. Moreover, the elevated cellular BCAAs levels induced by glucose exposure in HepG2 cells were also reversed by ECHS1^K101R^ overexpression (Supplementary Fig. 8b). These results were all consistent with BCAAs levels being regulated by the AcK101 controlled activity of ECHS1.

In support of the idea that BCAAs are activators of mTOR signaling^50^, accumulated in response to ECHS1 inactivation, the mTOR signaling pathway, was activated by *ECHS1* knockdown (Fig. 7e), but inactivated by ECHS1 overexpression (Fig. 7f), as determined by detecting the phosphorylation levels of the ribosomal protein S6 kinase (P-S6K) on T398. Moreover, while supplemental leucine, isoleucine and valine all activate mTOR, ECHS1 overexpressing in HEK293T cells diminished isoleucine or valine’s ability, but only partially suppressed leucine’s ability to activate mTOR (Supplementary Fig. 8c), confirming that BCAAs, in particular isoleucine and valine accumulation, contribute to ECHS1 downregulation-induced mTOR activation. P-S6K levels were also elevated in hepatocytes from *Sirt3* ^*−/−*^ mice and in *SIRT3* knocked down HEK293T cells (Fig. 7g, Supplementary Fig. 8d), and the increase in P-S6K levels induced by *SIRT3* knockdown could be partially abolished by ECHS1^K101R^ overexpression (Supplementary Fig. 8d) or saturated by BCAAs supplementation to HEK293T cells (Fig. 7h), indicating that K101 acetylation-induced BCAA accumulation activates mTOR signaling. Moreover, *ECHS1* or *SIRT3* knockdown in Chang liver cells resulted in larger cell sizes (Fig. 7i), as assayed by fluorescence-activated cell sorting, and facilitated cell proliferation (Fig. 7j), assessed by measuring the percentage of cells in G2/M phase, under nutrient starving conditions. Both effects could be abolished by rapamycin treatment (Fig. 7i, j), confirming that ECHS1 inactivation promotes cell growth by activating mTOR signaling.

### Nutrients induce FA accumulation

**Fig. 8:**
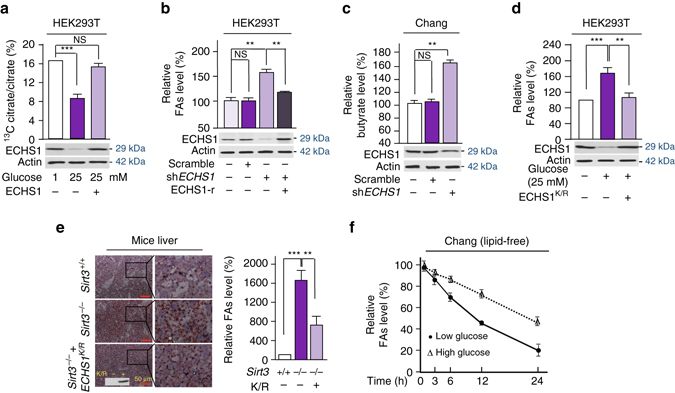
Nutrient induces FAs accumulation. **a** The conversion of ^13^C-palmitate to ^13^C-citrate was traced in HEK293T cells cultured in low and high glucose media, and in ECHS1 overexpressing HEK293T cells cultured in high glucose media. **b** Relative FAs levels in HEK293T cells, *ECHS1* knocked down HEK293T cells and *ECHS1* knocked down HEK293T cells overexpressing *shRNA* resistant ECHS1 (ECHS1-r) were detected. All levels were normalized to the total proteins. **c** The relative butyrate levels in Chang liver cells and *ECHS1* knocked down Chang liver cells were determined (*n* = 4). The butyrate levels in untreated Chang liver cells (25 μM) were set as 100%. **d** The relative FAs levels in HEK293T cells cultured in low glucose (−), high glucose (+), and high glucose with ECHS1^K101R^ overexpression were determined (*n* = 3). **e** The FAs levels in the liver of wild type, *Sirt3* ^*−/−*^, and *ECHS1* ^*K/R*^ overexpressing *Sirt3* ^*−/−*^ 129/C57BL6 mice were assessed by oil red staining. Four mice of each group were analyzed. Relative FAs levels were determined by comparing intensity of signals, *Sirt3* ^*+/+*^ value was set as 100% arbitrarily (*right*). **f** The relative (to time 0) intracellular FAs levels in Chang cells that were maintained in high glucose (25 mM) and low glucose (1 mM) were detected (*n* = 3) at time points as indicated. For all figures, mean values with SD are reported. NS not significant; *P < 0.05; **P < 0.01; ***P < 0.001

Consistent with glucose inactivating ECHS1, HEK293T cells exposed to high glucose utilize less palmitate, traced by ^13^C-labeling^48^, but overexpression of ECHS1 reversed glucose inhibition to palmitate utilization (Fig. 8a). These were in line with that *ECHS1* knockdown in HEK293T cells leading to an ∼40% increase in total cellular FAs (Fig. 8b), and *ECHS1* knockdown in Chang cells led to a 65% increase in cellular butyrate, a short chain fatty acid (Fig. 8c). Moreover, the increase of FAs in HEK293T cells by ECHS1 knockdown was reversed by introducing shRNA resistant ECHS1 into the cells (Fig. 8b). The FAs accumulation induced by 25 mM glucose exposure in HEK293T cells was also reversed by ECHS1^K101R^ overexpression (Fig. 8d). These facts confirmed that the intracellular FAs levels are subject to regulation by ECHS1. In line with the fact that Sirt3 deacetylates and activates ECHS1, the accumulation of FAs in the liver of *Sirt3* ^*−/−*^ mice was partially rescued by forced expression of non-acetylable ECHS1^K101R^ in mice (Fig. 8e, Supplementary Fig. 9). Moreover, high glucose concentrations which induces ECHS1 acetylation, slows down cellular fatty acids decrease when Chang cells were cultured in fatty acids depleted media (Fig. 8f). These results are all in line with the notion that nutrients block FAs oxidation through acetylating K101 on ECHS1.

### Nutrients induce apoptosis resistance

**Fig. 9:**
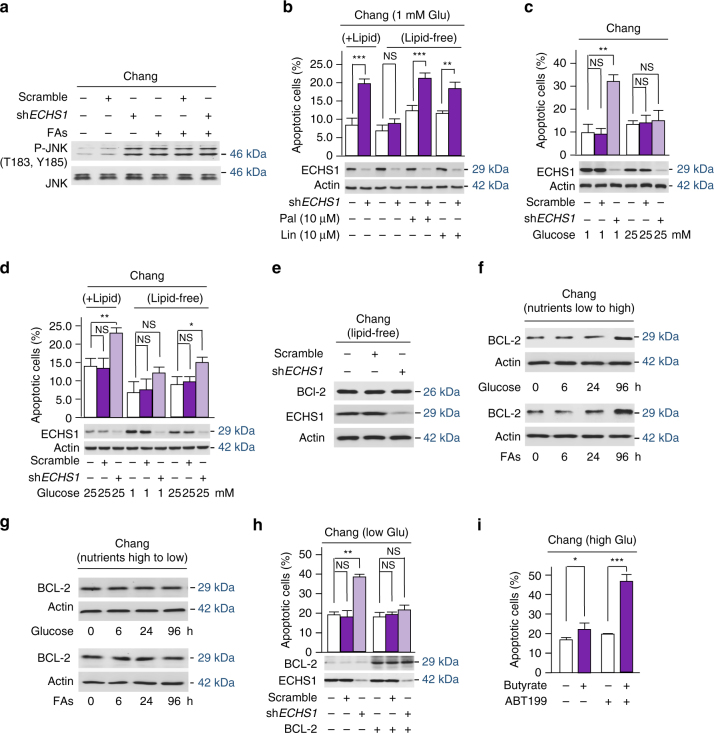
Nutrient induces apoptosis resistance. **a** The p-T183 and p-Y185 levels of JNK were analyzed for their responses to ECHS1 knockdown in Chang cells cultured in DMEM media with and without fatty acids supplementation. **b** The apoptotic rates of Chang and ECHS1 knockdown Chang cells that were cultured under presence and absence of lipids, and lipid-free media supplemented with saturated palmitate or unsaturated linoleic acid, were determined (*n* = 4). **c** The percentages of apoptotic Chang cells and apoptotic *ECHS1* knocked down Chang cells were determined when cells were cultured in the presence of 1 mM and 25 mM glucose (*n* = 4). **d** The apoptotic rates of Chang and ECHS1 knockdown Chang cells that were cultured under presence and absence of lipids, and lipid-free media supplemented with low and high glucose, were determined (*n* = 4). **e** BCL-2 levels in Chang cells and ECHS1 knockdown Chang cells that were cultured in lipid-free media were compared. **f** Chang cells were conditioned in glucose- or fatty acids-free media for 24 h before they were transferred to media containing high glucose (25 mM) or high fatty acids (250 nM), BCL2 levels were monitored over time. **g** Chang cells were conditioned in high glucose or high fatty acids for 96 h before they were transferred to low glucose (1 mM) or low fatty acids (250 nM) media, BCL2 levels were monitored over time. **h** The apoptotic rates in Chang cells and ECHS1 knockdown Chang cells cultured under low glucose were determined under with and without BCL-2 overexpression. **i** Chang cells were cultured in the presence of 25 mM glucose. The percentages of apoptotic cells of untreated and 1 mM butyrate-treated cells with or without ABT-199 treatment were determined (*n* = 4). For all figures, mean values with SD are reported. not significant; *P < 0.05; **P < 0.001; ***P < 0.001

Nutrient-induced accumulation of FAs such as butyrate in cells would induce lipotoxicity and lipoapoptosis^51, 52^. Indeed, knockdown of ECHS1 mildly activated Jun N-terminal kinase (JNK) signaling, a readout of lipoapoptosis, in a fatty acids saturable manner (Fig. 9a). Additional evidence that lipoapoptosis accounts for nutrient-induced apoptosis is that depleted fatty acids from the culture media abolished ECHS1 knockdown-induced apoptosis, while adding back excessive fatty acids, in particular the saturated fatty acid palmitate, resulted in high levels of apoptosis and potentiated the apoptosis promoting ability of ECHS1 knockdown in Chang cells cultured in 1 mM glucose media (Fig. 9b). Similarly, although ECHS1 knockdown rendered Chang cells cultured in high glucose (25 mM) had higher basal apoptotic rate than those cultured in glucose (1 mM) (Fig. 9c, *empty bars*), consistent with the idea that high glucose induces apoptosis^11, 12^, depleting fatty acids from the culture media abolished the apoptotic promoting ability of ECHS1 knockdown in Chang cells that were cultured in both low glucose and high glucose (Fig. 9d), in line with a model that nutrients induce fatty acids accumulation in cells and cause lipoapoptosis.

Remarkably, cells continuously exposed to high glucose were resistant to *ECHS1* knockdown-induced apoptosis (Fig. 9d), demonstrating that high glucose concentrations may induce apoptotic resistance. Given that nutrient exposure resulted in a higher expression of the anti-apoptotic factor BCL-2^53, 54^, we explored the possible underlying mechanism of this phenomenon. *ECHS1* knockdown in Chang cells cultured in low glucose lipid-free media failed to induce BCL-2 overexpression (Fig. 9e). Chang cells that were switched from low glucose (1 mM) and lipid-free media to high glucose or lipid rich (250 nM) media resulted in time-dependently increased BCL-2 (Fig. 9f). However, Chang cells cultured in high glucose and fatty acid media, when switched to low glucose or fatty acid media, resulted in negligible changes in expression of BCL-2 even after 96 h (Fig. 9g). Moreover, BCL-2 overexpression prevented ECHS1 depletion-induced apoptosis in Chang cells cultured under low glucose concentrations (Fig. 9h). Furthermore, selectively inhibiting BCL-2 by ABT199^55^ abolished Chang liver cells to develop apoptotic resistance under high glucose (Fig. 9i). These results are all consistent with a scenario in which rich nutrients impose selective pressure for BCL-2 overexpressing and apoptotic resistant cells in response to lipoapoptosis induced by rich nutrients.

### ECHS1 hyperacetylation is common in cancers

As metabolic organs are directly exposed to high levels of glucose and other nutrients, we predicted that hyperacetylated and inactivated ECHS1 accumulated BCAAs and FAs; therefore, increased BCL-2 expression would be observed in cancers originating from these organs. Fresh samples and histopathology slides of renal cell carcinoma (RCC), hepatocellular carcinoma, (HCC) and colon cancer (Supplementary Tables 1–3), which had clear boundaries between cancer and normal tissues were utilized, enabling us to compare differences between cancer tissues and adjacent normal tissues. The western blot analyses indicated that the expression levels of ECHS1, but not other analyzed β-oxidative enzymes, were severely decreased in all three cancers (Supplementary Fig. 10a). These findings were consistent with the immunohistochemistry (IHC) analyses performed on these cancers that showed a downregulation of ECHS1 in RCC, HCC, and colon cancer compared to their corresponding adjacent normal tissues (Supplementary Figs. 11–13). However, the change in the levels of ECHS1 were much more moderate in cancers such as prostate or ureter transitional cell carcinoma than in the cancers derived from metabolic organs (Supplementary Fig. 14). Thus, ECHS1 levels in cancer cells derived from metabolic organs are more sensitive to nutrient regulation than are other carcinomas (Supplementary Fig. 1).

**Fig. 10:**
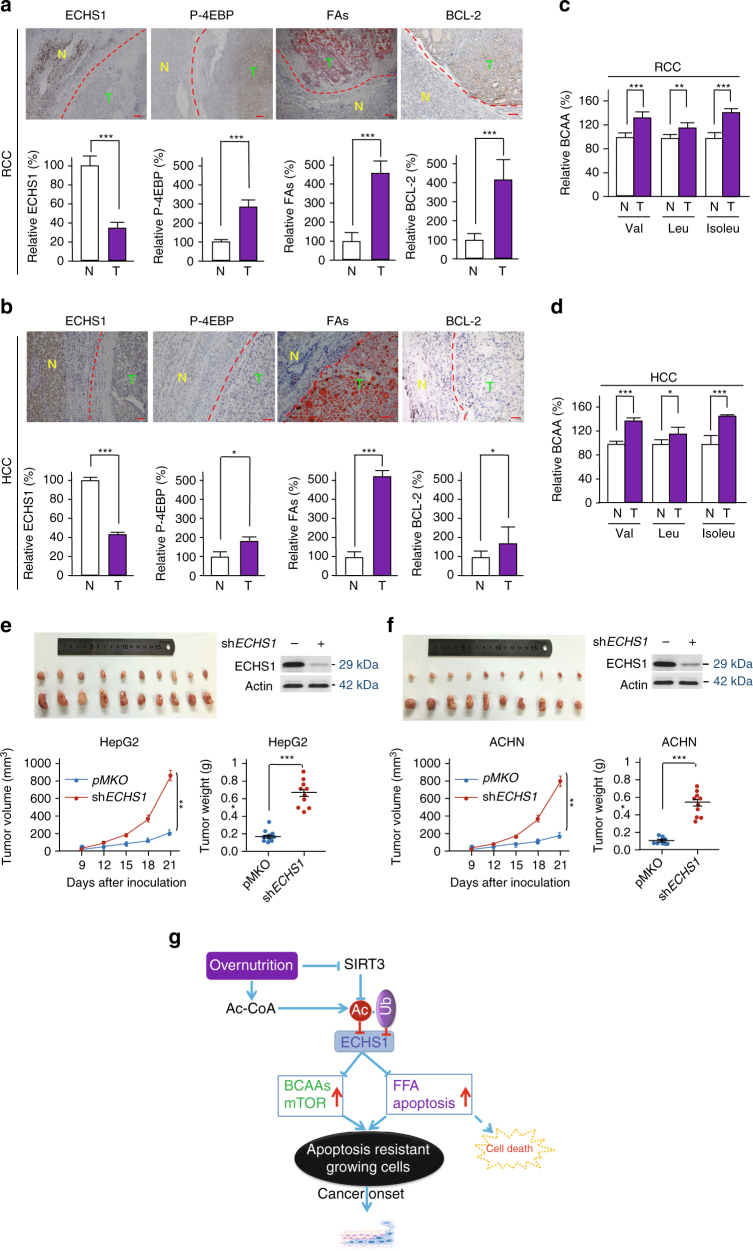
ECHS1 downregulation promotes BCAA and FA accumulation and cancer growth. **a**, **b** IHC analysis for ECHS1, P-4EBP, FAs, and BCL-2 in RCC (**a**, *scale bars*: 200 μm) and HCC (**b**, *scale bars*: 50 μm) specimens and their respective adjacent normal tissues. **c**, **d** Relative levels of BCAAs were determined in RCC (**c**) and HCC (**d**) tissues and their respective adjacent normal tissues. The levels of BCAAs in normal tissues were set as 100%. **e**, **f** The xenograft tumor growth of HepG2, ECHS1 knockdown HepG2, ACHN and ECHS1 knockdown ACHN cells were analyzed in *Balb/c* nude mice (4–6 weeks old). **g** Schematic diagram indicating how rich nutrients enrich ECHS1-inactivated cells and promote cancer initiation. Nutrients inactivate ECHS1 cause accumulation of BCAAs and FFAs, which induce mTOR activation and lipoapoptosis, respectively. The latter resulted in selection for apoptosis resistant cells and facilitate cancer transformation. Mean values with SD are reported. *P < 0.05; **P < 0.01; ***P < 0.001

The pathologic link between ECHS1, AcK101 acetylation, mTORC1 activation, and apoptotic resistance was supported by the significantly lower ECHS1 levels in cancer tissues and by IHC analyses with AcK101 antibody staining that resulted in similar signals between tumor and adjacent normal tissues (Supplementary Fig. 15), suggesting that ECHS1 is hyperacetylated in tumor tissues. Consistent with this prediction, western blot assays for affinity-purified endogenous ECHS1 found that ECHS1 from cancer tissue was acetylated to a greater degree than was normal adjacent tissues corresponding to the tumor (Supplementary Fig. 10b). Increased BCAAs and FAs levels (Fig. 10a–d) were a consequence of ECHS1 inactivation; increase in the levels of phospho-4EBP, readout of mTOR signaling activation and BCAAs accumulation; higher BCL2 levels, a signature of apoptosis resistance, were all increased in fresh tissue samples of RCC and HCC (Fig. 10a, b, Supplementary Figs. 16–21).

### ECHS1 hyperacetylation promotes cancer growth

Moreover, ECHS1 knockdown in HepG2 (Fig. 10e) and ACHN cells (Fig. 10f) both resulted in facilitated tumor xenografts growth in mice, and putting back K/Q mutant into ECHS1 knockdown HepG2 and ACHN cells resulted in faster tumor xenografts growth than those of putting back the wild type and K/R mutant (Supplementary Fig. 22). These results collectively confirmed the role of acetylation-induced ECHS1 downregulation in promoting tumor growth.

## Discussion

Elucidation of nutrients sensory mechanism shed light on understanding the pathologic mechanism of nutrient deregulation-associated disease. In the current study, we provided evidence to support the notion that ECHS1 senses nutrients through K101 acetylation. Overnutrition promotes cancer initiation by providing intracellular conditions that foster transformation of cancer cells. ECHS1 determines the metabolic fates of BCAAs and FAs. Under nutrient-limiting conditions, FAs and BCAAs, the two most important energy reservations for cells, are directed to catabolism to supply energy to the cells, requiring the activity of ECHS1. When nutrients are abundant and the energy supply is sufficient, ECHS1 is effectively inactivated by nutrient-promoted K101 acetylation, which downregulates ECHS1 through synergized effects of ECHS1 inactivation, blockade of ECHS1 mitochondria translocation, and facilitated ECHS1 degradation. BCAAs and FAs are thus directed to the anabolic metabolism to facilitate the generation of anabolic signals mTOR and the formation of membrane, respectively, in cells. This ingenious design ensures the survival of cells under both nutrient-limiting and nutrient-rich conditions. Prolonged inactivation of ECHS1 such as constant exposure to nutrients; however, may result in cancer initiation. Nutrients induce the accumulation of BCAAs in cells and provide cells with anabolic signals (mTOR). Moreover, nutrient abundance results in the accumulation of fatty acids, while facilitating membrane generation in cells, it exerts lipo-toxicity that induces cellular apoptosis. This imposes selection pressures on the cells. Those cells that were resistant to apoptosis for example, cells with higher BCL-2 expression, will be enriched by overnutrition. These two nutrients-induced consequences, if persistent, will increase the number of apoptotic resistant cells, which are then easily transformed to cancer cells (Fig. 10g). Therefore, we propose over-nutrition increases cancer onset by providing selective conditions that allow the survival of cancerous cells. Our model is echoed by our findings that, in cancers such as RCCs, HCCs and colon cancers, which originated from metabolic organs that are exposed to nutrients, ECHS1 is severely downregulated. Furthermore, reports that diabetes and obesity, which are both associated with low levels of ECHS1 but high levels of glucose and/or FAs, increased risk of cancers^56^ while caloric restriction, a state of low nutrients, delays cancer onset^57^, is consistent with our hypothetical model.

Compared to normal cells which depend on nutrient signals to inactivate ECHS1 and to trigger the anabolic metabolism, cancer cells may trigger anabolic metabolism by gaining mTOR activating mutations or ECHS1 inactivating mutations. The anabolic metabolism-promoting effects of ECHS1 inactivation are supported by the fact that either homozygous or compound heterozygous mutations in ECHS1 induce lactic acidosis^58^, a signature of the Warburg effect and anabolic metabolism activation. Moreover, ECHS1 downregulation is accompanied by increased apoptosis levels^59^, in line with our observation that ECHS1 downregulation activates apoptosis. Furthermore, supporting that GCN5 inactivates and SIRT3 activates ECHS1, GCN5 is known to promotes cancer cell growth^60^ and SIRT3 is a confirmed tumor suppressor that suppresses tumor development through maintaining mitochondrial integrity under stress^61, 62^. Therefore, our data suggests that suppression of mTOR activation and a reduction in the apoptotic resistance may overcome the tumorigenic effects of overnutrition and its associated link to diabetes and obesity. This hypothesis is supported by the evidence that mTOR inactivation by rapamycin and BCL-2 inactivation by ABT-99 both maintain anticancer properties^3, 55^. Together, alternative anticancer measures may be possible for overnutrition and its associated metabolic diseases by activating ECHS1.

## Methods

### Reagents and antibodies

Commercial antibodies used in this study and their manufactures were: α-Flag (cat. no. F3165, 1:3000, Sigma-Aldrich), α-Actin (cat. no. A00702, 1:5000, Genscript), α-ECHS1(cat. no. H00001892-D01P, 1:1000, ABNOVA), α-ECHS1 for IP (cat. no. H00001892-PW1, 1:50, ABNOVA), α-p70 S6 Kinase (cat. no. 9202, 1:1000, Cell Signaling Technology.), α-Phospho-p70 S6 Kinase (Thr389) Antibody (cat. no. 9205, 1:1000, Cell Signal Technology), α-CPT1A (cat. no. ab176320, 1:1000, abcam), α-CPT2(cat. no. ab18114, 1:1000, abcam), α-ACADL (cat. no. 17442-1-AP, 1:1000, proteintech), α-EHHADH (cat. no. sc-393123, 1:500, Santa Cruz), α-HADHA (cat. no. sc-292195, 1:1000, Santa Cruz), α-ACAA2 (cat. no. ab128929, 1:1000, abcam), α-SOD2 (cat. no. GTX116093, 1:1000, GeneTex), α-TOM40 (cat. no. sc-365467, 1:1000, Santa Cruz), α-SIRT3 (cat. no. ab137689, 1:1000, abcam), α-GAPDH (cat. no. ab1289915, 1:1000, abcam), α-COX IV (cat. no. 4850, 1:1000, Cell Signaling Technology), α-GFP (cat. no. m20004m, 1:5000, Abmart), α-SAPK/JNK (cat. no. 9252, 1:1000, Cell Signaling Technology), α-Phospho-SAPK/JNK (Thr183/Tyr185) (cat. no. 9251, 1:1000, Cell Signaling Technology), α-BCL2 (cat. no. A0208, 1:1000, ABclonal). α-HA, α-pan-acetyllysine, and α-K101 specific-acetyllysine antibodies were generated during this study by immunizing rabbits with synthetic antigens at Shanghai Genomic Inc. and AB Mart Shanghai Inc. Protein-G-Agarose (16-266) was from Millipore, α-FLAG M2 Affinity Gel (A2200) was from Sigma-Aldrich. TSA was from Cell Signal Technology, MG-132 was purchased from Merck, Free Fatty Acid Quantification Kit was purchased from BioVision, NAM and all other chemicals used were from Sigma-Aldrich.

### Cell culture and treatments

HEK293T cells (ATCC Number: CRL-11268), 786-O(ATCC Number: CRL-1932), ACHN renal cell cancer cells (ATCC Number: CRL-1611), SMMC7712 (CBP60210, Shanghai Cell Bank), Huh7 liver cancer cells (CBP60202, Shanghai Cell Bank), HCT 116 (ATCC Number: CCL-247), SW620 colon cancer cells (ATCC Number: CCL-227), LNCaP (ATCC Number: CRL-1740), PC3 prostate cancers cells(ATCC Number: CRL-1435), Chang cells (ATCC Number: CCL-13) were used in this study.

All cell lines were tested and authenticated by morphology and growth rate, and were mycoplasma free. Cells were cultured in either normal Dulbecco’s Modified Eagle’s Medium (DMEM) (HyClone) supplemented with 10% newborn bovine serum (NCS) (Biochrom), 100 U/ml penicillin (Invitrogen) and 100 μg/ml streptomycin (Invitrogen) or conditioned medium made from DMEM base (Sigma-Aldrich) as specified. Cell transfection was performed using PEI or calcium phosphate methods. Deacetylase inhibitor treatments were carried out by adding TSA (0.5 μM, final concentration) and NAM (5 mM, final concentration) to the culture medium 18 and 5 h before harvesting, respectively. To alter the nutrients of cultured cells, DMEM base (Sigma) was used as the media base. Glucose was added in to the media base at final concentrations of either 1 or 5 mM as specified to mimic low glucose, and at 25 mM to mimic high glucose, respectively. Equal molar of glutamate and aspartate (Sigma) was added to the media to final concentrations as indicated to constitute different levels of amino acids. Equal molar of linoleic acid and palmitic acid (Sigma) was added to the media to final concentrations as indicated to constitute different levels of fatty acids. Linoleic acid or palmitic acid was individually added into the media base at indicated final concentrations to mimic levels of unsaturated and saturated fatty acids supplemental, respectively.

### Clinical samples

All clinical cancer samples were obtained from Shanghai Cancer Center, Fudan University with the signed consent from the patients and approval of the ethics committee of Fudan University.

### Mouse primary hepatocytes

Primary mice hepatocytes were isolated from adult mice by a modified collagenase method^63^. Briefly, the cells were plated in M199 medium supplemented with 100 U/ml penicillin^64^, 100 μg/ml streptomycin, 10% fetal bovine serum, 500 nM dexamethasone (dex; Sangon Biotech), 10 nM insulin (Actrapid, Novo Nordisk), at a density of 2.5 × 10^5^ cells per well on six-well plates or 1 × 10^5^ cells per 24-well plates. After attachment (3–4 h), hepatocytes were maintained in M199 medium with antibiotics and 100 nM dexamethasone for 16 h before used for further manipulations.

### Immunoprecipitation and western blotting

Unless specified, collected cells were lysed in 0.5% NP-40 buffer containing 50 mM Tris-HCl (pH 7.5), 150 mM NaCl, 0.5% Nonidet P-40 and a mixture of protease inhibitors (Sigma-Aldrich). After centrifugation at 12,000 rpm 4 °C for 15 min, the supernatant of lysate was collected and incubated with anti-Flag M2 agarose or other antibody beads for 3 h at 4 °C. The beads were washed three times with lysis buffer before boiling with SDS loading buffer. For Co-IP experiments, cells were lysed in 0.1% NP-40 buffer containing 50 mM Tris-HCl (pH 7.5), 150 mM NaCl, 0.1% Nonidet P-40 and a mixture of protease inhibitors. Endogenous ECHS1 immunoprecipitation was carried out by lysing cultured cells or cells extracted from tissues with 0.5% NP-40 buffer, followed by incubated lysate with anti-ECHS1 antibody and Protein-G-Agarose beads at 4 °C for 4–5 h, followed by the standard immunoprecipitation and immunoblotting procedures.

Detection of acetylation and other proteins by western blotting was achieved by using 50 mM Tris (pH 7.5) with 10% (v/v) Tween 20 and 1% peptone (OXOID) as blocking buffer and dilute primary and secondary antibodies in 50 mM Tris (pH 7.5) with 0.1% peptone26,65.

Important uncropped western blots are supplemented as Supplementary Fig. 23.

### Tandem affinity purification

HEK293T cells were transfected with pMCB-SBP-Flag-ECHS1 or pMCB-SBP-Flag, each containing a puromycin-resistance selection marker. The stable cell lines were established by selecting with 5 μM puromycin. The ECHS1 stable cells were lysed on ice with 0.1% NP-40 buffer containing protease inhibitors cocktail for 30 min. Cell lysate was incubated with Flag beads for 3 h followed by elution with Flag peptide. The resulted solution was incubated with streptavidin-Sepharose beads (GE Healthcare) for 3 h at 4 °C, and the beads were washed three times with NP-40 buffer followed by three times with 50 mM NH_4_HCO_3._On-beads tryptic digestion were carried out at 37 °C overnight. The resulted peptides in supernatant were dried in Concentrator Plus (Eppendorf) before subject to mass spectrometry analysis.

### Oil red staining

Oil red staining was performed as previous described^65^. Briefly, slides with specimens were brought to room temperature and washed in running water to remove OCT compound (Tissue Tek 4583). Slides were placed in 50% isopropanol for 3 min in 100% isopropanol for a further 3 min and stained with 0.5% ORO (O-0625, Sigma-Aldrich) in 100% isopropanol for 2 h. The slides were then differentiated in 85% isopropanol for 3 min for three times, followed by washing with running water, stained with Mayer’s hematoxylin for 15 s and bluing in running water for a further 10 min. The slides were mounted with Glycerol Jelly Mounting Medium (Beyotime) before subject to analyzing.

### Immunohistochemistry

Tissue sections were prepared from the formalin-fixed paraffin embedded specimens.

Antigen retrieval of cancer specimens was performed by incubating the slides in 10 mM citrate buffer (pH 6.0) at 99 °C for 10 min. The endogenous peroxidase activity was inactivated in solution of methanol with 3% H_2_O_2_. The quantification of IHC results were performed by an experienced pathologist. The intensity was calculated according to positive areas and positive degree. Sections were staining with respect antibodies (1:2000) using a Dako Envision kit (DAKO).

### Flow cytometry

Different stable cell lines cultured on purpose were digested with Trypsin, washed two times with PBS, and fixed with 70% ethanol overnight. The following day, the fixed cell clusters were washed two times with PBS. The cell samples were incubated with 400 μl RNase for 10 min at room temperature against light. After removing buffer, 400 μl propidium iodide (Sigma) was added to incubate with the cell samples for a further 10 min at room temperature against light. Finally the cell suspension was filtered by Nylon sieve and subjected to Flow Cytometry analysis. Cell sizes were analyzed by forward side scatter (FSC) and proliferation rate was determined by measuring percentage of cells in the G2/M phase.

### Gene silencing

Small RNA interference (*si*) and stable knockdown were used. Stably shRNA knockdown cells were made by co-transfecting cells with pCMV-VSV-G, pCMV-Gag-Pol and shRNA plasmids by the calcium phosphate method. DMEM containing 10% NCS was used to culture the cells for 6 h after the transfection. After 24 h of transfection, supernatant of the cultured medium was collected and used as retrovirus preparation to infect cells at a density of 10% confluent in 90 mm-diameter dishes. Cells were re-infected 48 h after the initial infection, using 5 μg/ml puromycin (Amresco) for selecting cells. The knock-down efficiency of the stable cell lines was verified by q-PCR or western blotting.

Sequences used for gene silence in this study:

*ECHS1*: GCTTGCCATGATGTGTGATAT

*SIRT3*: TCTTGCTGCATGTGGTTGATT

*SH3RF2:* GACCTTCCTCAAGGACGATAT

*GCN5*: GCTCTACACAACCCTCAAATT

### Ubiquitinylation assay

For ubiquitinylation analysis, Protease inhibitor MG132 was added 4 h before harvest. Cells were collected and lysed directly in 1% SDS buffer (Tris-HCl pH 7.5, 0.5 mM EDTA, 1 mM DTT) followed by boiling for 10 min immediately after lyses. Immunoprecipitation of lysed proteins was carried out by adding antibodies to 10× diluted lysis solution with 10 mM Tris-HCl buffer.

### In vitro acetylation assay

ECHS1-Flag were incubated with GCN5-Myc and acetyl-CoA in HAT buffer (50 mM Tris-HCl, pH 8.0, 5% glycerol, 0.1 mM EDTA, 50 mM KCl, 1 mM DTT, 1 mM PMSF, 10 mM sodium butyrate) in a final volume of 30ul for 30 min at 30 °C. Acetylated ECHS1-Flag were visualized by western blot.

### In vitro deacetylation assay

Recombinant SIRT3 was isolated from *E. coli (DE3)*. Synthetic AcK101-containing ECHS1 peptide (H-Gly-Ala-Asp-Ile-Lys^Ac^-Glu-Met-Gln-Asn-OH) or recombinant ECHS1 was used as substrate. Deacetylation reaction buffer includes: 30 mM HEPES (pH 7.0), 1 mM NAD^+^, 1 mM DTT, 0.6 mM MgCl_2_ and 0.5 mM PMSF. Products of deacetylation reactions were subject to MS analysis 1 h after the reaction was incubated at 37 °C.

### Expression and purification of recombinant ECHS1

Expression and purification of recombinant human WT, K101R mutant, K101Q mutant of ECHS1 was achieved by cloning signal sequence-free matured ECHS1 (28-290 a.a) and its mutants into pET-22b(+). Proteins were overexpressed in *E. coli*. BL21(*DE3*) induced with 0.5 mM IPTG when O.D._600_ of culture medium reached 0.6. The cells were harvested by centrifugation, sonicated and purified by a standard Ni-NTA purification procedure using HisTrap HP (GE Healthcare). The eluted protein was then centrifuged by Amicon Ultra-15 (Millipore, USA) to removing imidazole and stored in 50 mM Tris-HCl containing 20% glycerol at −80 °C for further experiments.

### ECHS1 enzyme assay

ECHS1 enzyme activity was assayed according to published methods^66^. Briefly, ECHS1 was added to a reaction mixture containing 130 μl 50 mM Tris-HCl (pH 8.0) and 0.25 mM crotonyl-CoA. The progress of the reaction was monitored by measuring decrease in absorbance at 263 nm.

### FAs and BCAAs analysis

Fatty acids levels were measured with Free Fatty Acid Quantification Colorimetric/Fluorometric Kit (Biovision), following the manufacturer’s instructions. BCAAs levels were measured by GC–MS. Briefly, cells were harvested with pre-chilled 80% methanol, 1 mM Ribitol was added to the lysate as an internal standard. After 8 h the samples were dried and derivatized consecutively with 1% methoxyamine hydrochloride/pyridine (70 °C for 1 h) and 20% N-tert-Butyldimethylsilyl-N-methyltrifluoro-acetamide/pyridine (37 °C for 0.5 h) before subject to assay using Agilent 6890-5973 GC–MS system.

### Targeted citrate formation assay

About 5 × 10^5^ cells were seeded and cultured in complete medium overnight and incubated in medium with or without glucose for 4 h. Leucine-1,2-^13^C2 (2 mM) or Palmitic acid-^13^C16(500 nM) was added 1 h before the cells were harvested for metabolite extraction with 1 ml of 4:1 v/v MeOH/H_2_O, which was pre-equilibrated at −80 °C. The extract and cells were collected into 1.5 ml eppendorf tubes and centrifuged for 10 min at 5000 g in solvent, with the resulting supernatant being evaporated using a speed-vac. Samples were re-suspended in 200 μl buffer (20 mM ammonium hydroxide/20 mM ammonium acetate (pH = 9.0) in 95:5 water:acetonitrile) and citrate levels were determined by targeted liquid-chromatography tandem mass spectrometry (LC–MS/MS). Ratios of ^13^C-labeled over unlabeled citrate were used to show the utilization of leucine or palmitatic acid.

### Animals

All animal procedures were performed in accordance with the approval of the animal care committee at Fudan University. The *Sirt3* KO mice were generated by employing Cre-LoxP recombination technology, and their genotyping was confirmed by PCR^67^. All mice were housed on a 12:12 h light:dark cycle at 25 °C. Experiments were performed using 6- to 8-month male littermates. Mice were randomized into each of the groups. Samples were blindly processed during the course of the experiments, and the outcome was assessed. Naked plasmid injections were performed via the tail vein, with 100 μg plasmid DNA per mouse. Livers were removed and whole-cell homogenates were made with 0.5% NP-40 buffer and used for western blotting. For oil red staining, live tissues were placed OCT (Tissue Tek 4583) in a peel-away mold and frozen at −80 °C for further analysis.

### Statistics

Unless specified, results are expressed as mean ±SEM. Comparisons between groups were made by unpaired two-tailed Student’s *t*-test. Differences were considered statistically significant if *P*<0.05.

### Data availability

The authors declare that all the other data supporting the findings of this study are available within the article and its Supplementary Information files and from the corresponding authors upon reasonable request.

## Electronic supplementary material

Supplementary Information
