# Supplementary material for: Enoyl-CoA hydratase-1 regulates mTOR signaling and apoptosis by sensing nutrients
Source: Nat Commun. 2017 Sep 6;8:464. doi: 10.1038/s41467-017-00489-5 (PMC5587591; doi:10.1038/s41467-017-00489-5)
Supplement: Supplementary file 1 — Supplementary Information [file 41467_2017_489_MOESM1_ESM.pdf]

Description of Supplementary Files

File Name: Supplementary Information
Description: Supplementary Figures and Supplementary Tables

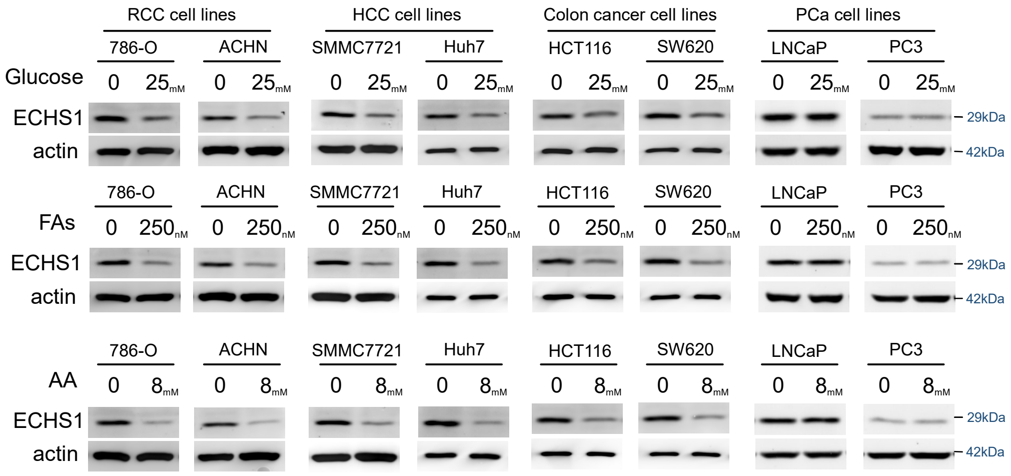

Supplementary Figure 1, Nutrients downregulate endogenous ECHS1 protein levels in cell lines derived from metabolic organs. The endogenous ECHS1 levels of 786-O and ACHN renal cell cancer cells, SMMC7712 and Huh7 liver cancer cells, HCT-116 and SW620 colon cancer cells and LNCaP and PC3 of prostate cancers were compared between these cells cultured under absence and presence of indicated concentrations of glucose, fatty acids and amino acids, respectively.

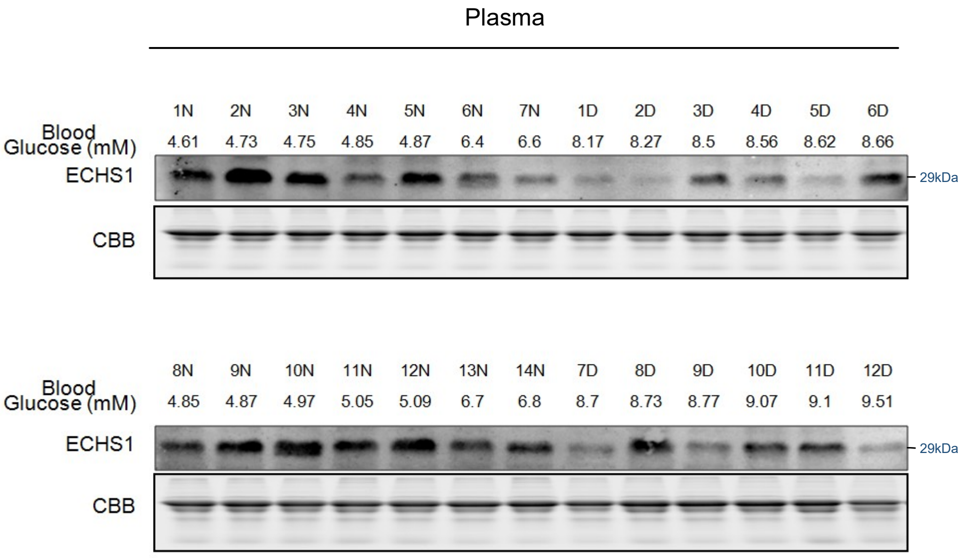

Supplementary Figure 2, Circulating ECHS1 levels were inversely correlated to blood glucose levels. ECHS1 levels of plasma of normal people (N, n=14) and untreated diabetes patients (D, n=12) were determined by western blots, ECHS1 levels were normalized to total protein levels (Coomassie Brilliant Blue staining, CBB). Relative ECHS1 levels were summarized in **Fig. 1f**.

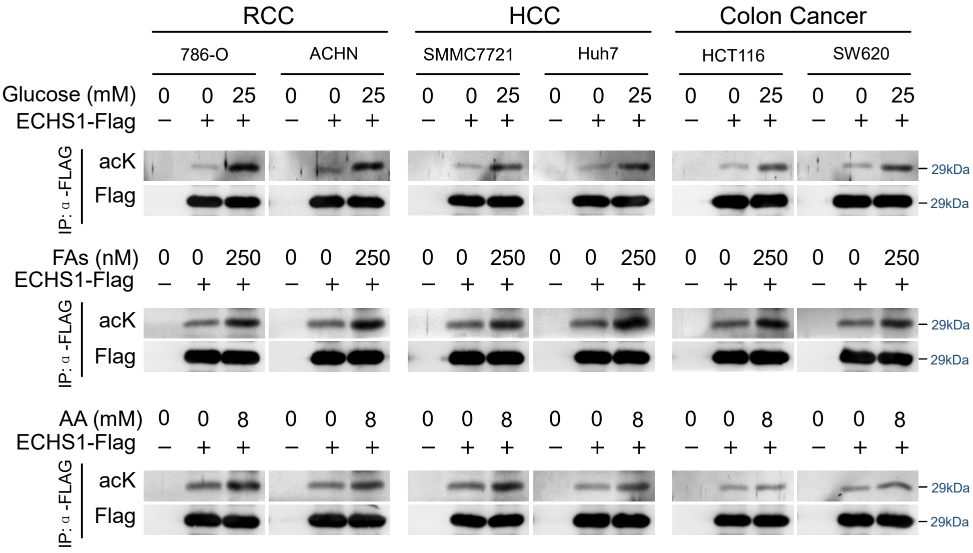

Supplementary Figure 3, Nutrients upregulate acetylation levels of ectopically expressed ECHS1 from various cultured cell lines. The ECHS1 acetylation levels of 786-O and ACHN renal cell cancer cells, SMMC7712 and Huh7 liver cancer cells and HCT 116 and SW620 colon cancer cells were compared between these cells cultured under absence and presence of indicated concentrations of glucose, fatty acids and amino acids, respectively. All acetylation levels were normalized to ECHS1 protein levels.

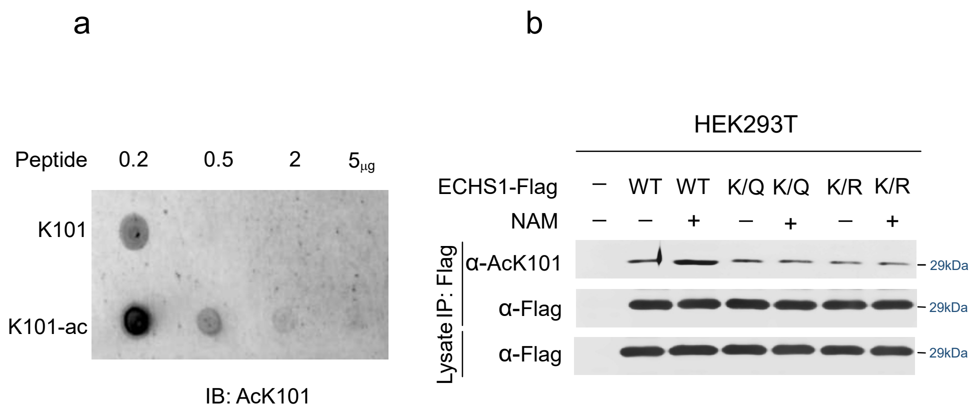

Supplementary Figure 4, Characterization of AcK101 antibody. The specificity and affinity of home-made AcK101 antibody was tested for its binding to (a) synthetic acetylated (K101-ac) and un-acetylated (K101) peptides, and to (b) wildtype, K101Q (K/Q) and K101R (K/R) ECHS1 expressed from HEK293T cells cultured at absence or presence of deacetylase inhibitor nicotinamide (NAM).

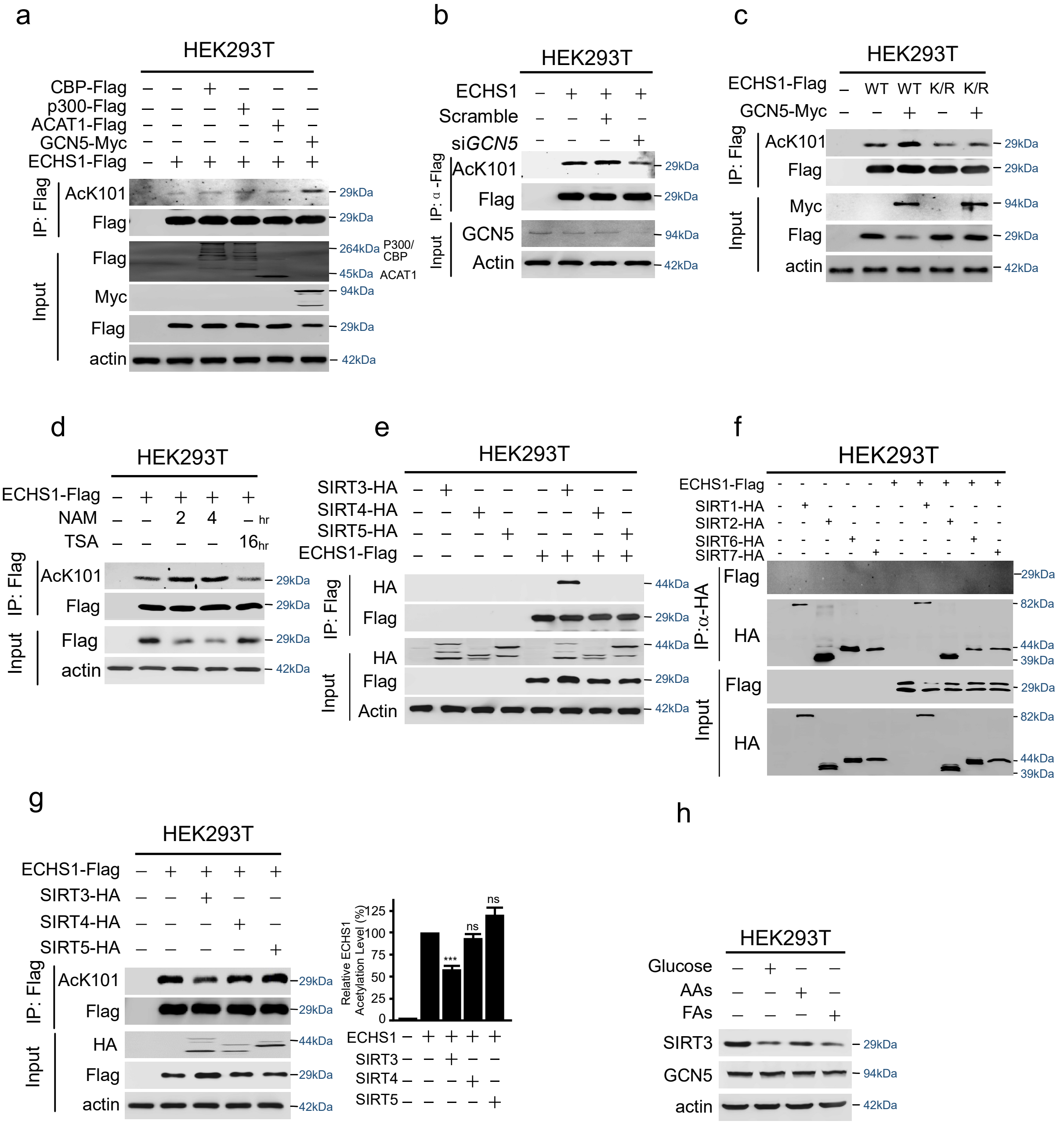

Supplementary Figure 5, GCN5 acetylates and SIRT3 deacetylates ECHS1. (a) The AcK101 levels were determined for ECHS1 expressed alone or co-expressed with CBP, P300, ACAT1, and GCN5, respectively, in HEK293T and the relative AcK101 levels were normalized to ECHS1 levels. (b) The AcK101 levels of ectopically expressed ECHS1 from HEK293T cells or *GCN5* knocked down HEK293T cells were determined. (c) The AcK101 levels of ECHS1 and its K/R mutant expressed alone or co-expressed with GCN5 in HEK293T cells were detected. (d)The AcK101 level of

ECHS1 purified from HEK293T cells treated with or without NAM or TSA for the indicated time were determined. (e) Flag-tagged ECHS1 was co-expressed with SIRT3, SIRT4, or SIRT5. The interaction between ECHS1 and SIRTs was determined by co-immunoprecipitation. (f) SIRT1, SIRT2, SIRT6 and SIRT7 didn't interact with ECHS1. Flag-tagged ECHS1 was co-expressed with HA-tagged cytosolic/nuclear SIRT1, SIRT2, SIRT6 and SIRT7, respectively. After SIRTs were affinity isolated from cells lysate with HA-beads, ECHS1 co-purified with each SIRT were detected by western blot employing Flag antibody. (g) The AcK101 levels of ECHS1 co-expressed with SIRT3, SIRT4, or SIRT5 were determined. (h) The expression levels of SIRT3 and GCN5 were detected in HEK293T cells that were cultured in DMEM and DMEM supplemented with glucose, fatty acids and amino acids, respectively.

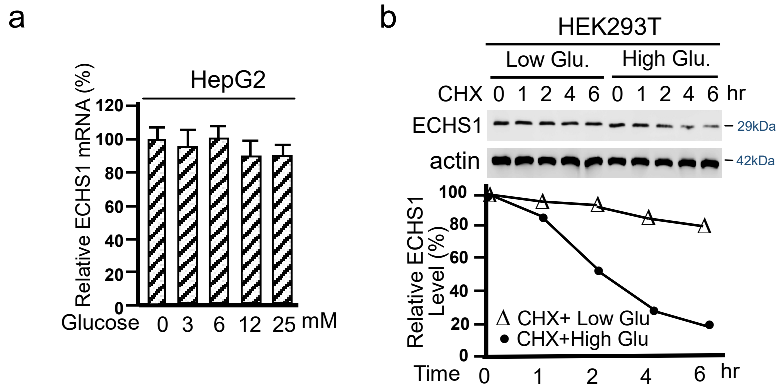

Supplementary Figure 6, Glucose promotes degradation of ECHS1. (a) HepG2 cells were cultured in DMEM media containing various level of glucose, the *ECHS1* mRNA levels were determined (n=3) for each culture. (b) HEK293T cells cultured under low- (1 mM) and high- (25 mM) glucose were treated with CHX before harvest. Endogenous ECHS1 levels in lysates of harvested cells were determined by western blots at time points as indicted after the lysis (upper panel). ECHS1 levels were normalized to respective actin levels (lower panel).

a

Reference	Score	Pep. Match	MW
ECHS1	845	1091	31367
BAZ2B	46	11	240311
CC146	33	7	112736
BRWD1	32	18	262756
WDR87	31	17	332959
**SH3RF2**	**27**	**7**	**79320**
BAT2	27	11	228721
PSMD1	26	7	105836
BRWD3	26	16	203598
ECM29	25	15	204160
PSMD3	24	37	60939

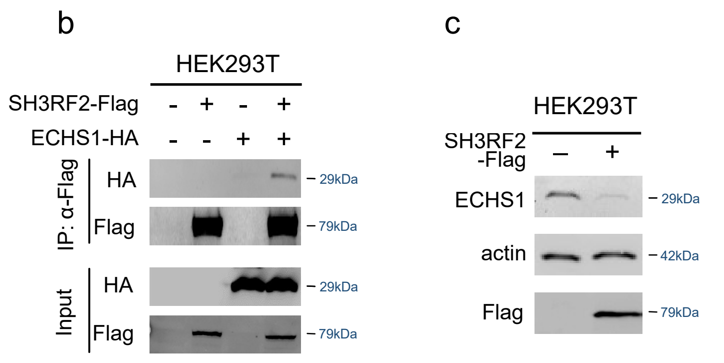

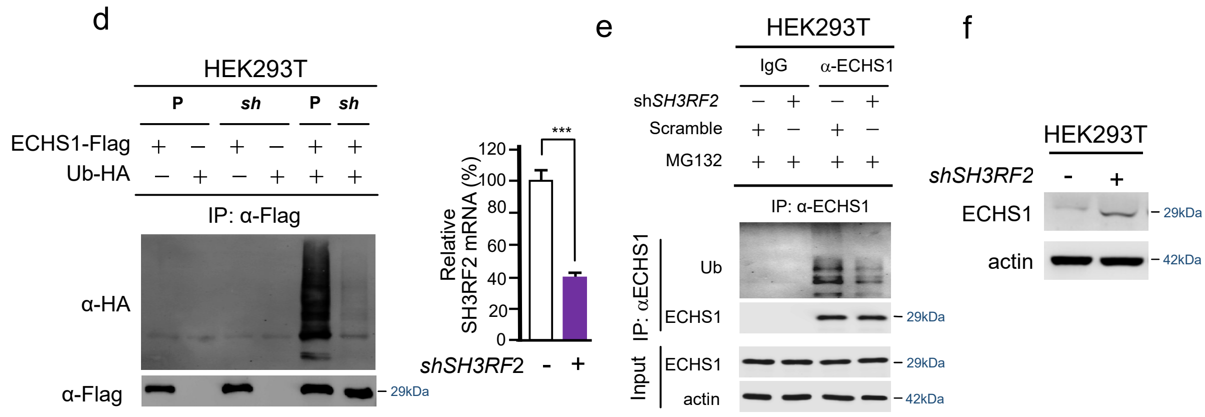

Supplementary Figure 7, SH3RF2 is the E3 ligase of ECHS1. (a) HEK293T cells stably express Flag- and SBP-tagged ECHS1 were harvested and lysed with NP-40 buffer and ECHS1 protein was purified with Flag beads. Flag peptides eluted ECHS1 from Flag beads was then purified again by streptavidin beads. Proteins in streptavidin were digested with trypsin on beads and the resulted peptides were subject to LC-MS/MS analysis. High probability proteins were shown in the list. (b) Flag tagged SH3RF2 and HA tagged ECHS1 were co-transfected into HEK293T cells. SH3RF2 proteins were isolated with Flag beads 48 hours after transfection and the co-IP of ECHS1 was determined by western blot. (c) The endogenous ECHS1 levels of HEK293T cells were assessed under with and without SH3RF2 overexpression. (d-e) The ubiquitinylation level of overexpressed (d) and endogenous (e) ECHS1 was detected in HEK293T and sh*SH3RF2* knockdown (*sh*) HEK293T cells. PMKO (P) control was employed. MG132 was added to the cell culture throughout the assays. (f) The endogenous ECHS1 levels were detected in HEK293T cells and *SH3RF2* knockdown HEK293T cells. Mean values with SD are reported. ***: $P< 0.001$.

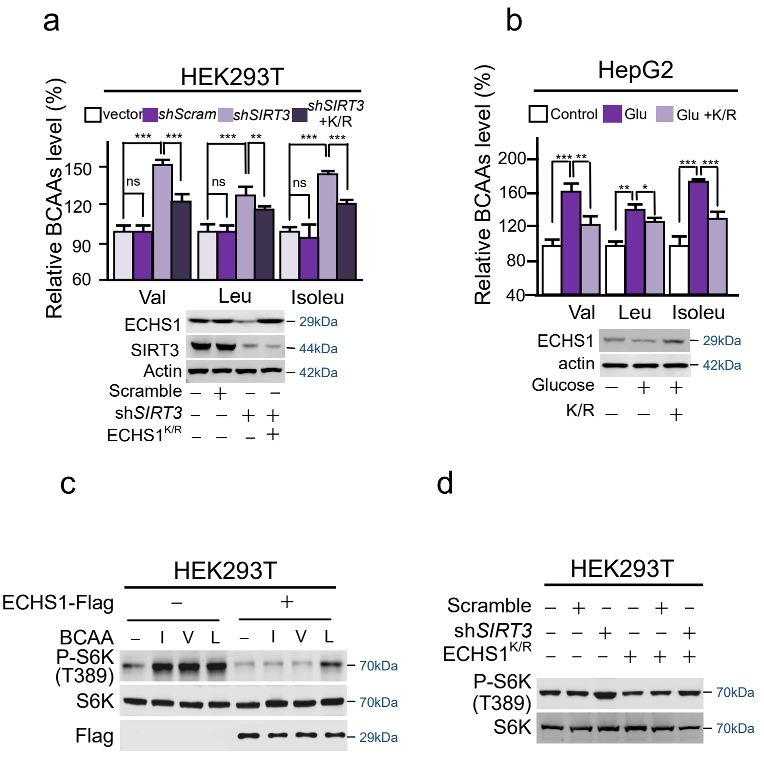

Supplementary Figure 8, ECHS1 inactivation activates mTOR signaling. (a) BCAAs levels of HEK293T cells, sh*SIRT3* knocked down HEK293T cells and sh*SIRT3* knocked down HEK293T cells overexpressing ECHS1^{K101R} were determined by GC-MS (n = 4). BCAA levels were normalized to those of HEK293T cells. (b) BCAA levels of HepG2 cells cultured in the presence of 1 mM glucose (-), HepG2 cells cultured in the presence of 25 mM glucose (+), and HepG2 overexpressing ECHS1^{K101R} cultured in 25 mM glucose were determined by GC-MS (n = 3). BCAA levels were normalized to those of control cells. (c) The levels of P-T389 on S6K were determined in HEK293T and ECHS1-overexpressing HEK293T cells that were cultured in media supplemented with isoleucine, valine and leucine, respectively. (d) The levels of P-T389 on S6K were determined in HEK293T and in *SIRT3* knockdown HEK293T cells with and without ECHS1^{K101R} overexpression.

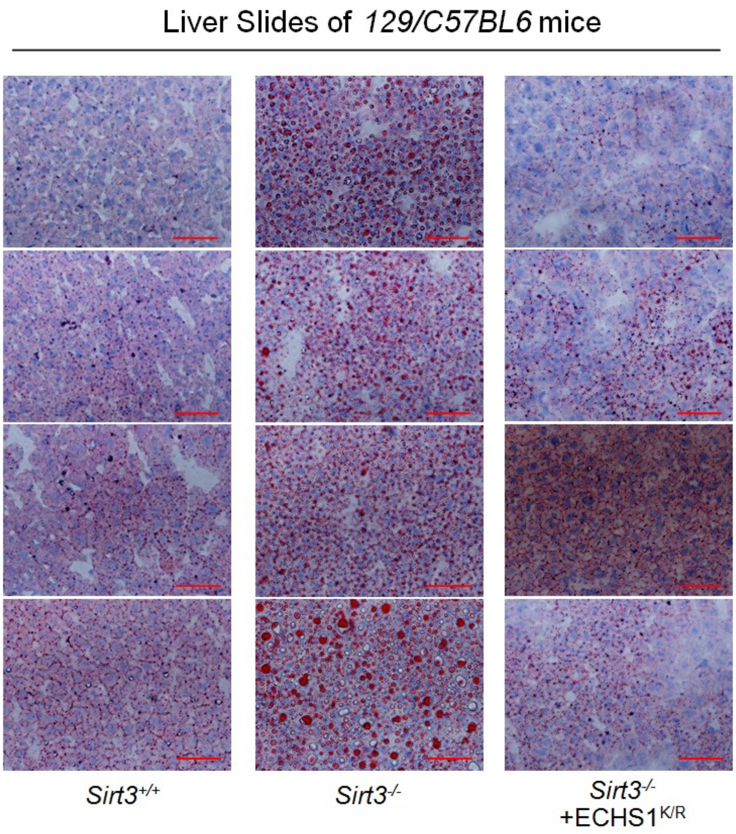

Supplementary Figure 9, *Sirt3* KO caused accumulation of FAs in hepatocytes of mice can be reversed by K/R mutant overexpression. Oil red staining was carried out to livers frozen sections of *129/C57BL6* mice, isogenic *Sirt3* $^{-/-}$ mice and *Sirt3* $^{-/-}$ mice forced to express K/R mutant. The intensity of Oil Red staining was quantified and the relative average values were presented with the FAs levels of wild type mice set as 100%.

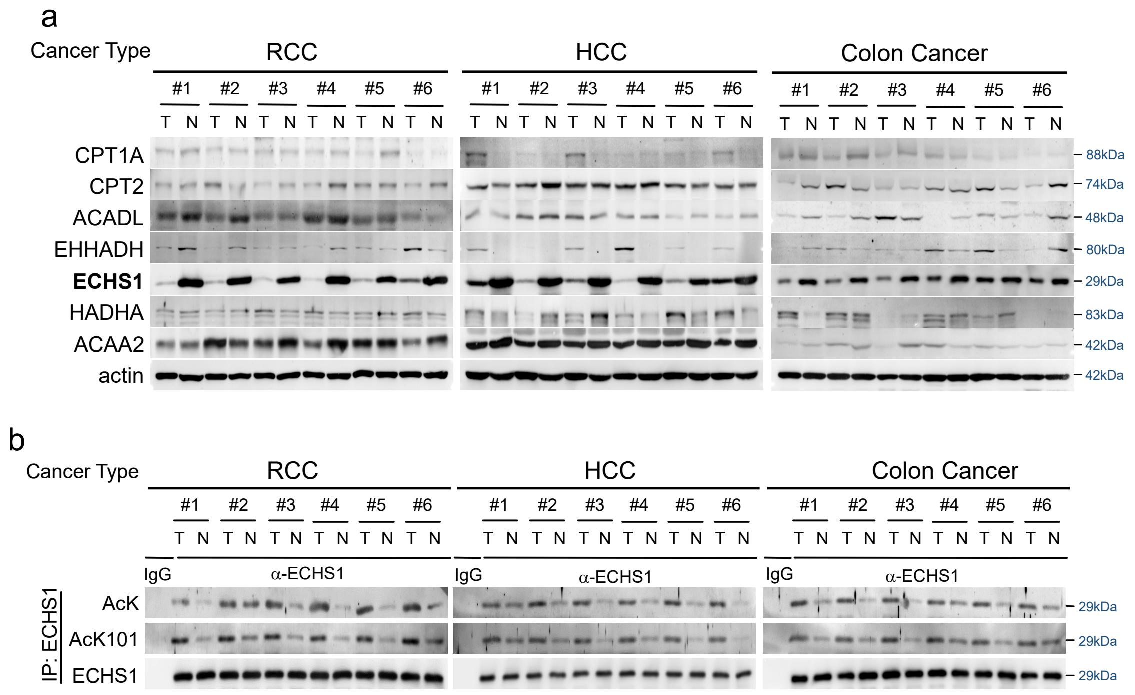

Supplementary Figure 10, ECHS1 hyperacetylation is common in cancers. (a) Western blot analysis for ECHS1 and other β-oxidation enzymes protein levels in RCC, HCC and colon cancer specimens and their respective adjacent normal tissues. (b) AcK and AcK101 levels of endogenous ECHS1 from RCC, HCC and colon cancer and their respective adjacent tissue were determined by western blot (n=6).

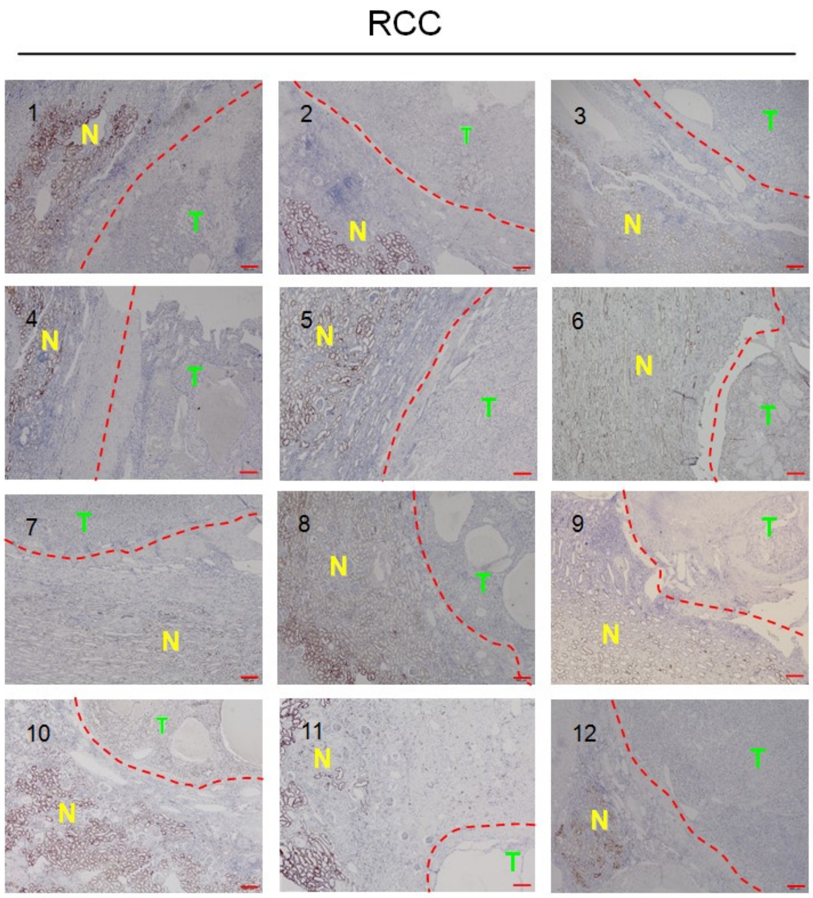

Supplementary Figure 11, IHC analysis for ECHS1 of RCC. Slides of RCC samples were subject to IHC analysis for ECHS1. Boundaries between cancer tissues and normal tissues were marked by red dashed lines. Scale of bars: 200 μm.

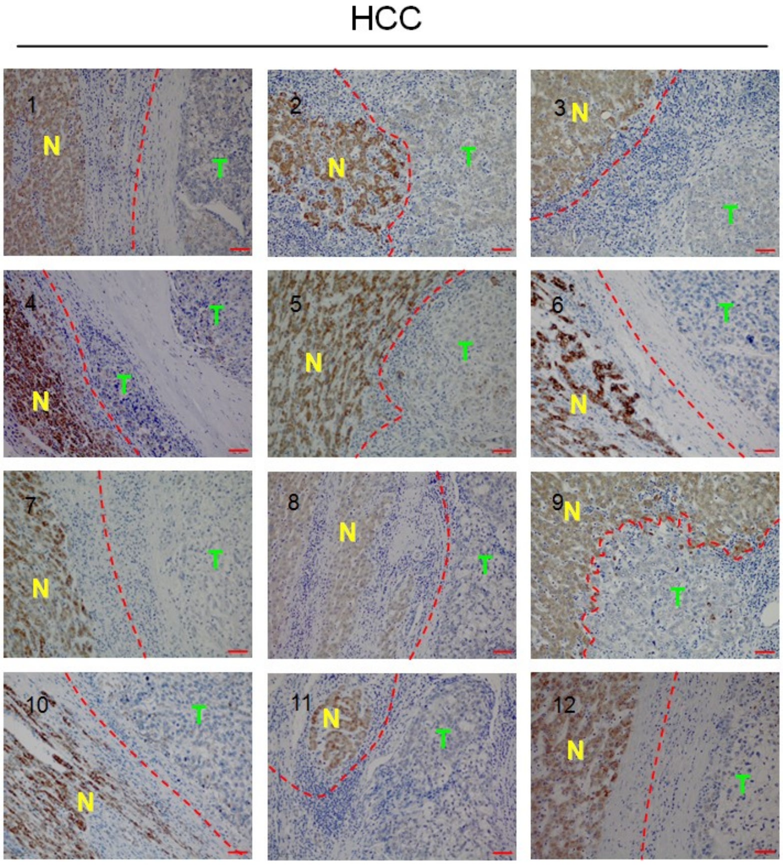

Supplementary Figure 12, IHC analysis for ECHS1 of HCC. Slides of HCC samples were subject to IHC analysis for ECHS1. Boundaries between cancer tissues and normal tissues were marked by red dashed lines. Scale of bars: 50 μm.

Colon Cancer

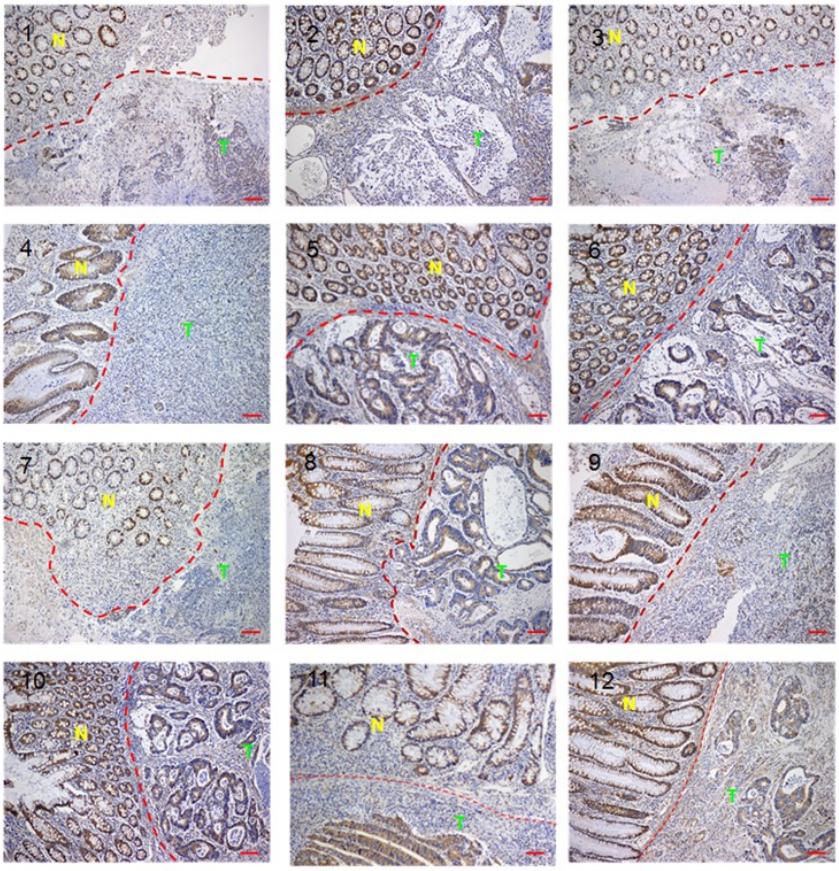

Supplementary Figure 13, IHC analysis for ECHS1 of colon cancer. Slides of colon cancer samples were subject to IHC analysis for ECHS1. Boundaries between cancer tissues and normal tissues were marked by red dashed lines. Scale of bars: 100 μm.

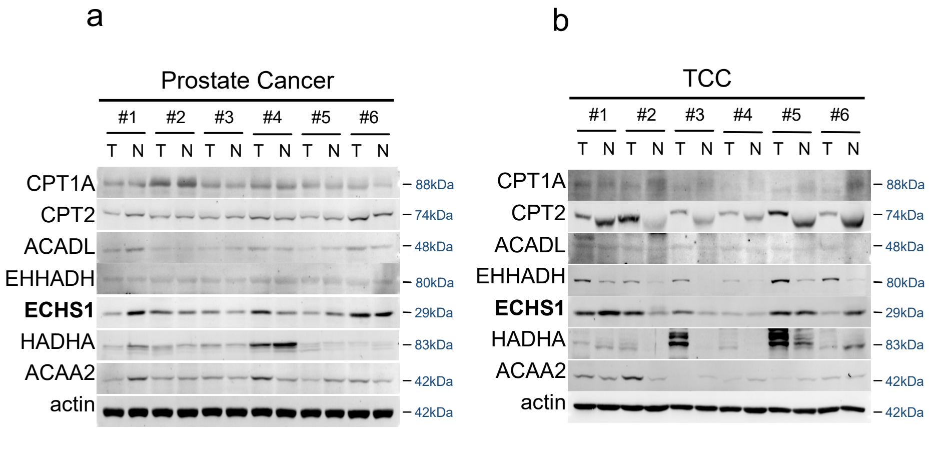

Supplementary Figure 14, ECHS1 levels are insignificantly differentially expressed in cancers of non-metabolic organs. Western blot analysis for ECHS1 and other β-oxidation enzymes protein levels in (a) prostate cancer and in (b) ureter transitional cell carcinoma (TCC) specimens and their respective adjacent normal tissues.

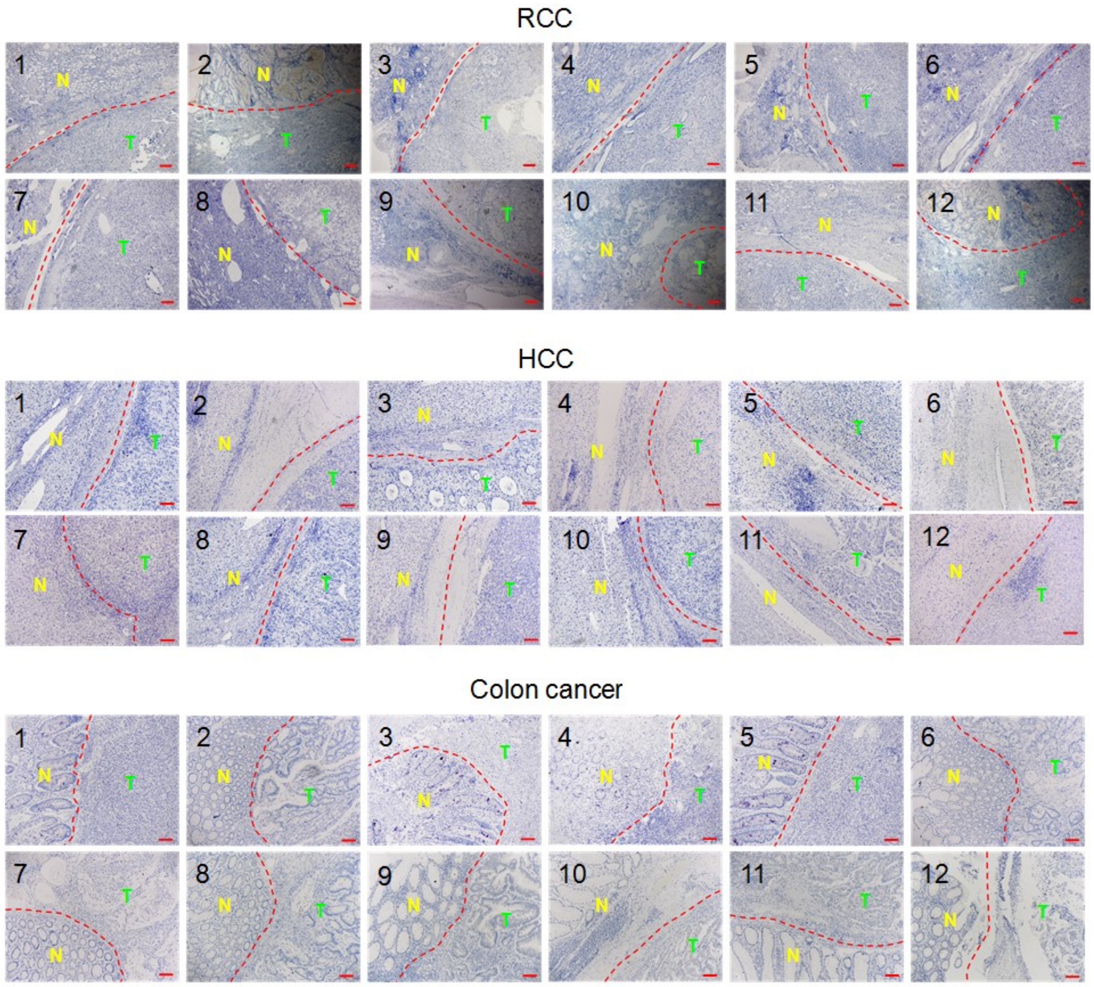

Supplementary Figure 15, IHC analysis for AcK101 in cancers. Slides of RCC, HCC and colon cancer samples were subject to IHC analysis for AcK101 of ECHS1. Boundaries between cancer tissues and normal tissues were marked by red dashed lines. Scale of bars: 200 μm for RCC, 100 μm for HCC and colon cancer.

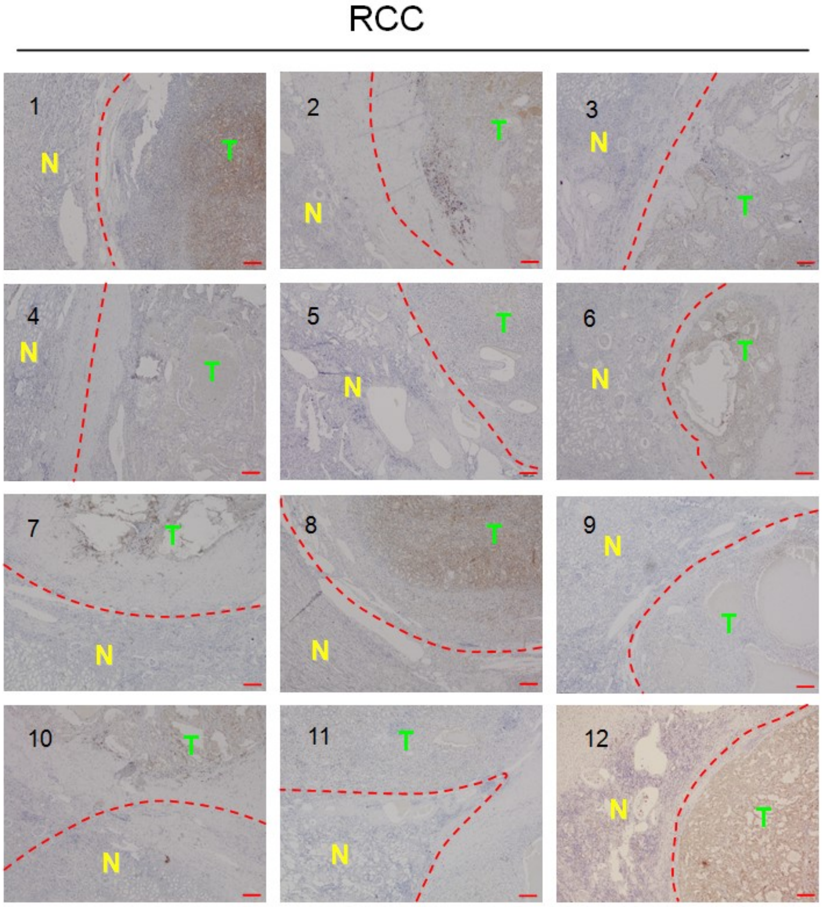

Supplementary Figure 16, IHC analysis for P-4EBP of RCC. Slides of RCC samples were subject to IHC analysis for P-4EBP. Boundaries between cancer tissues and normal tissues were marked by red dashed lines. Scale of bars: 200 μm.

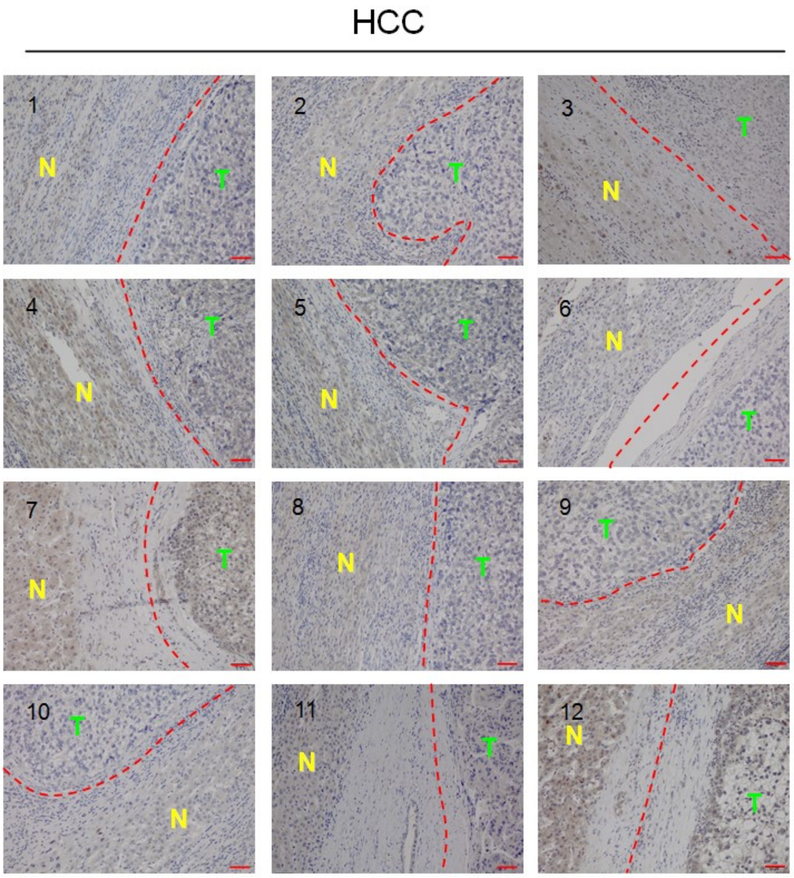

Supplementary Figure 17, IHC analysis for P-4EBP of HCC. Slides of HCC samples were subject to IHC analysis for P-4EBP. Boundaries between cancer tissues and normal tissues were marked by red dashed lines. Scale of bars: 50 μm.

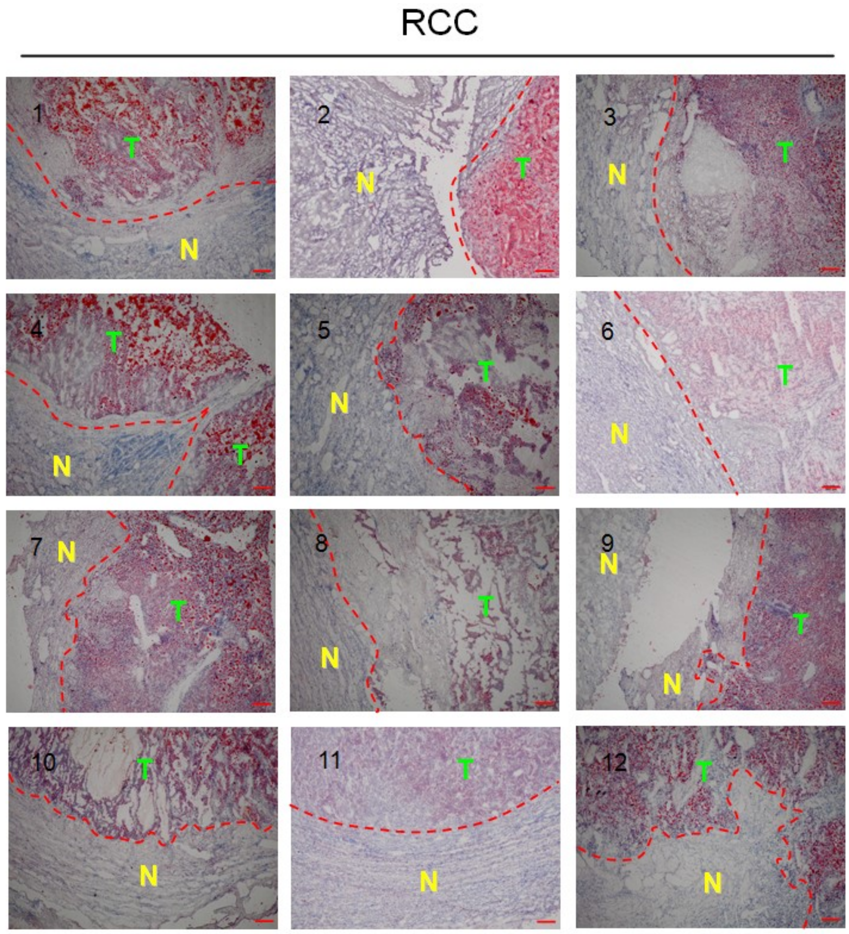

Supplementary Figure 18, Oil-Red staining of RCC. Slides of RCC samples were stained by Oil Red. Boundaries between cancer tissues and normal tissues were marked by red dashed lines. Scale of bars: 200 μm.

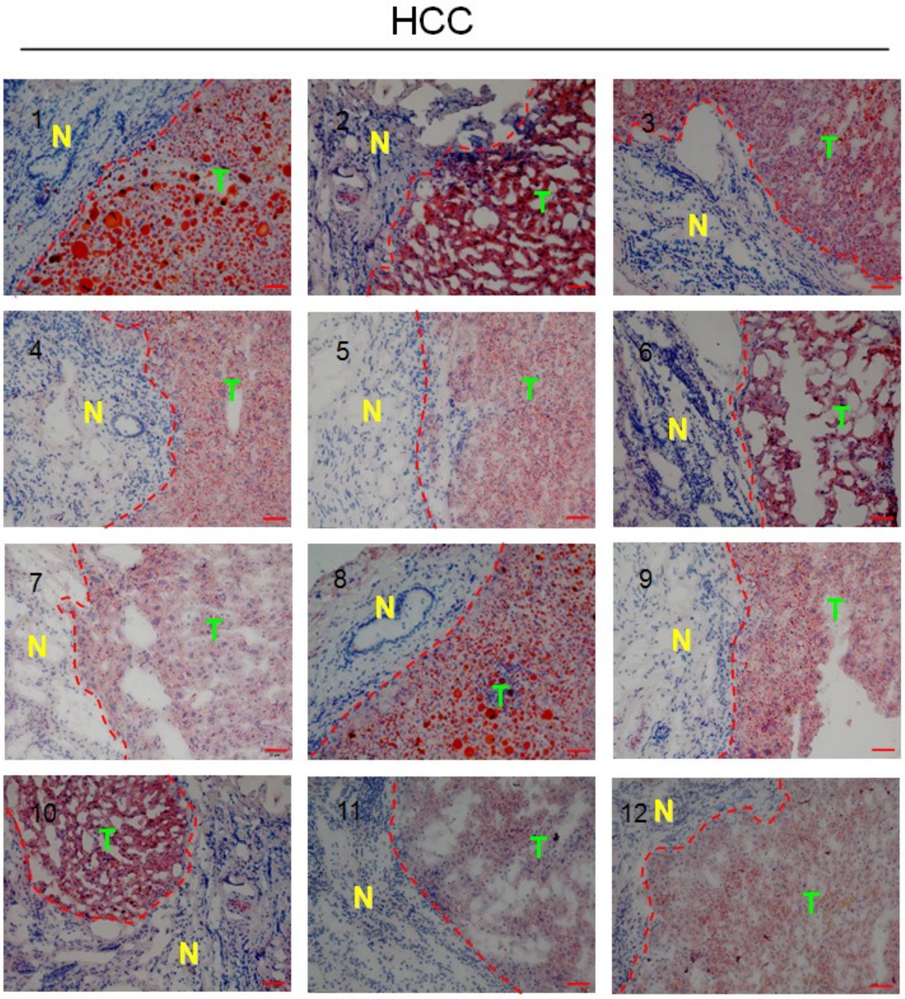

Supplementary Figure 19, Oil-Red staining of HCC. Slides of HCC samples were stained by Oil Red. Boundaries between cancer tissues and normal tissues were marked by red dashed lines. Scale of bars: 50 μm.

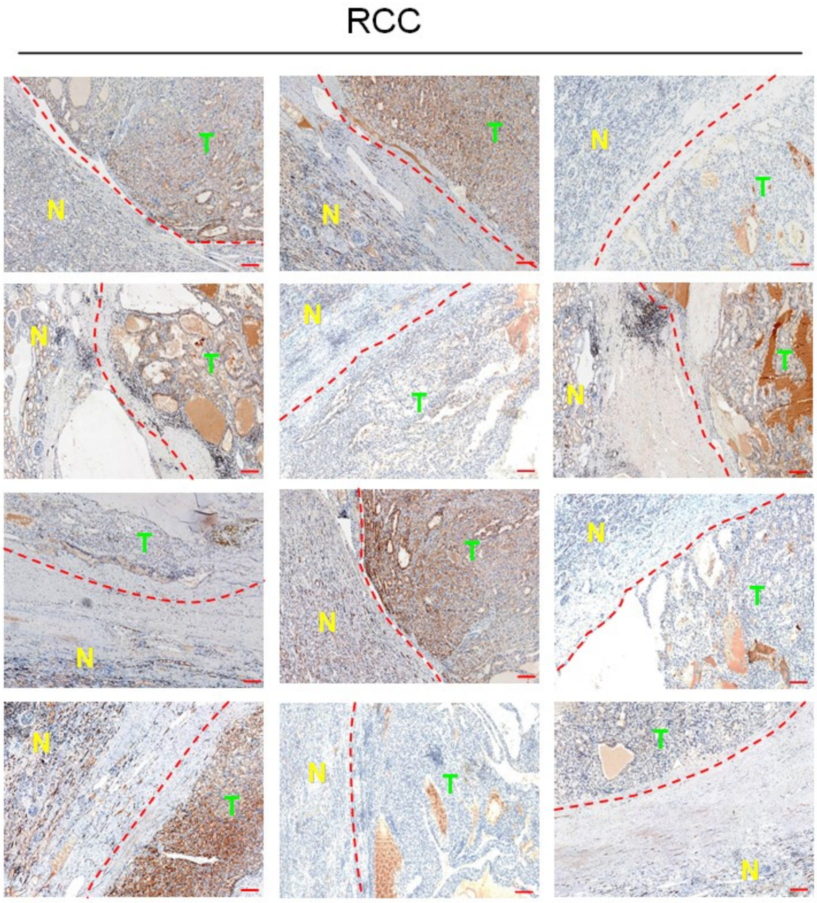

Supplementary Figure 20, IHC analysis for BCL-2 of RCC. Slides of RCC samples were subject to IHC analysis for BCL-2. Boundaries between cancer tissues and normal tissues were marked by red dashed lines. Scale of bars: 200 μm.

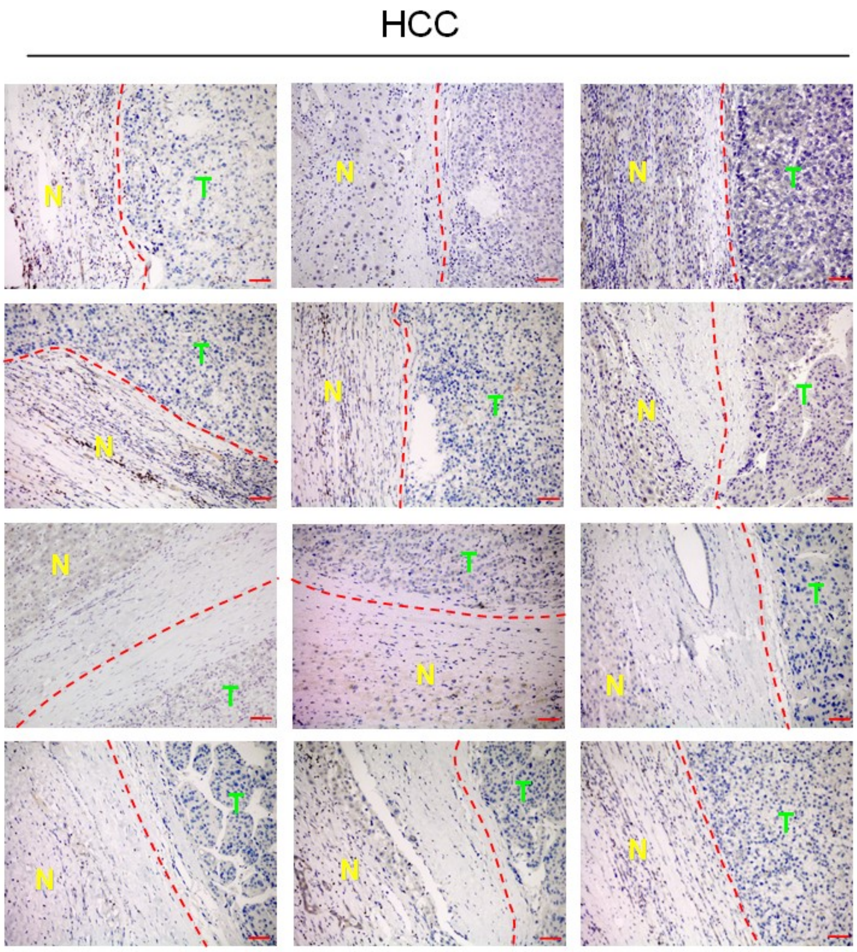

Supplementary Figure 21, IHC analysis for BCL-2 of HCC. Slides of HCC samples were subject to IHC analysis for BCL-2. Boundaries between cancer tissues and normal tissues were marked by red dashed lines. Scale of bars: 50 μm.

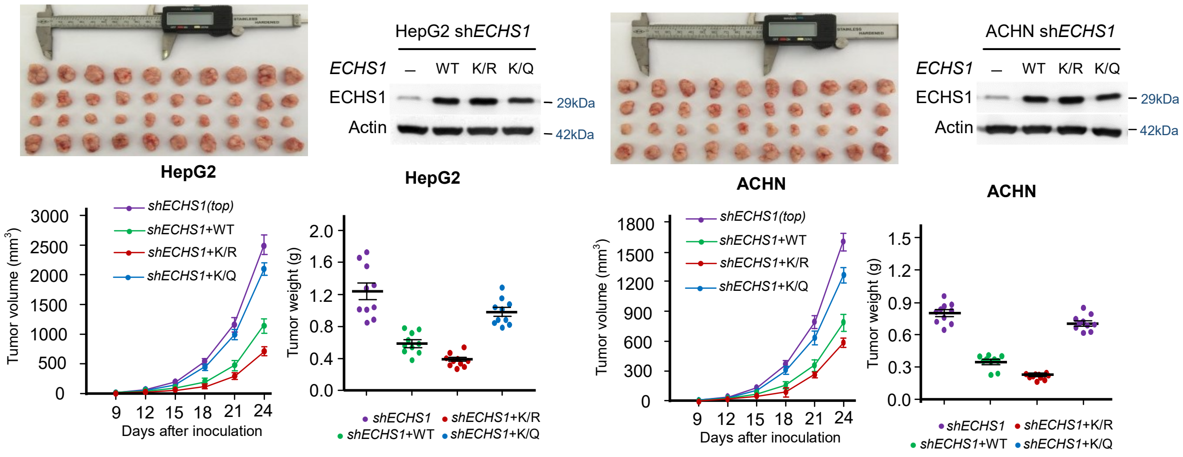

Supplementary Figure 22, Acetylation mimetic ECHS1 has higher potency to promote tumor growth. Wild type, K/Q and K/R mutant were put back at similar levels into HepG2 and ACHN cells with ECHS1 stably knockdown. The growth of xenografts of these cells was monitored by measuring both volume at times indicated and measuring weight 24 days after sacrificing. Mean values with SD are reported.

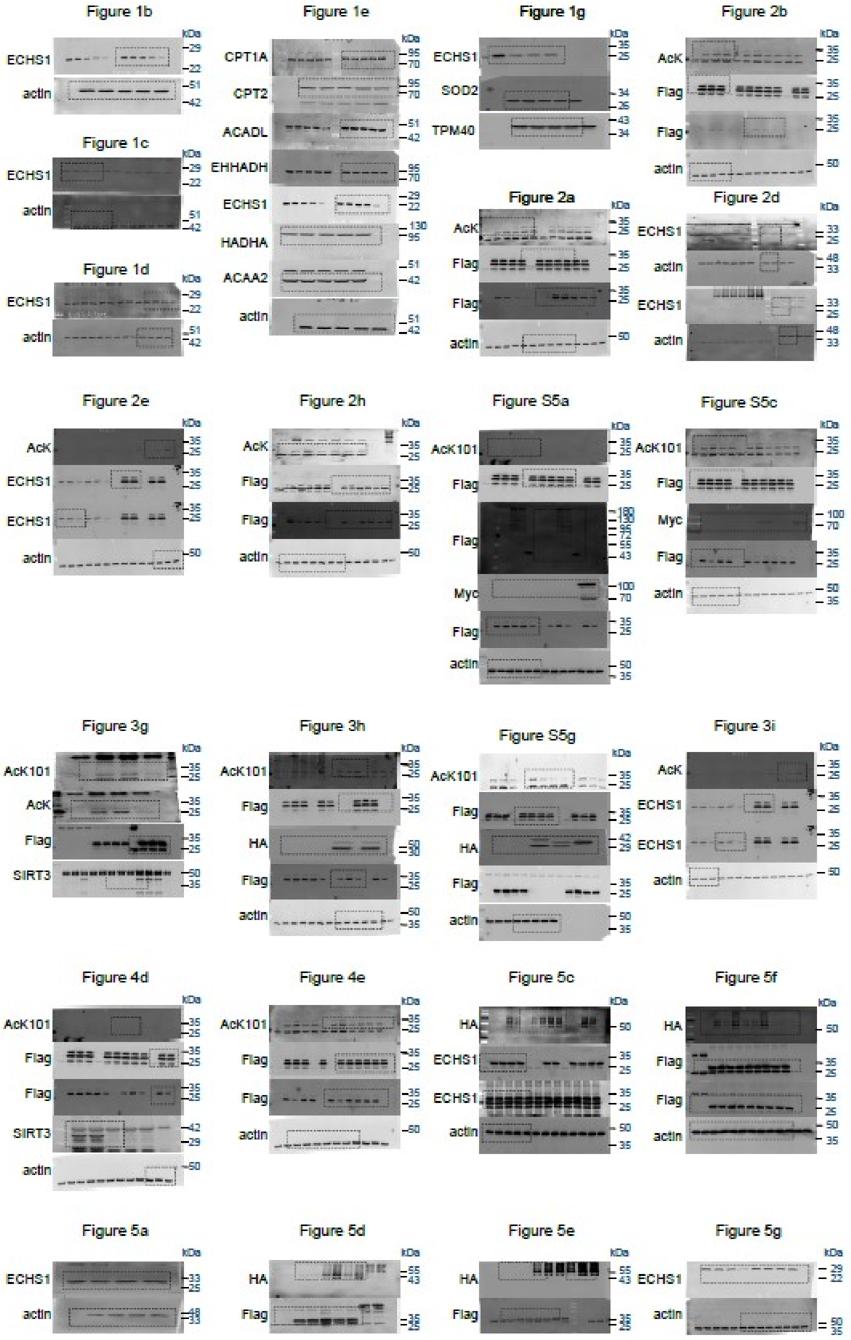

Supplementary Figure 23, Uncropped important Western Blots results. Data cropped from the gels are cycled in rectangles.

Sample ID	Age (years)	Gender	BMI (Kg/m^2)	Tumor site	Tumor size (cm)	Pathology	TNM stage	ISUP grade
**1**	45	Male	32.24	Right kidney	6.0×4.0×4.5	Clear cell RCC	T3N0M0	3
**2**	35	Male	24.42	Left kidney	3.5×3.5×3.0	Clear cell RCC	T1aN0M0	3
**3**	56	Female	25.85	Right kidney	2.5×2.5×2.0	Clear cell RCC	T1aN0M0	3
**4**	33	Female	18.26	Left kidney	4.5×3.5×3.0	Clear cell RCC	T1bNM0	1
**5**	37	Female	22.03	Right kidney	6.0×5.5×3.5	Clear cell RCC	T1bNM0	2
**6**	41	Male	25.83	Right kidney	4.5×4.0×3.5	Clear cell RCC	T1bN0M0	3
**7**	63	Male	30.10	Left kidney	5.5×5.0×3.5	Clear cell RCC	T1bN0M0	2
**8**	67	Male	23.88	Left kidney	5.5×4.6×4.2	Clear cell RCC	T1bN0M0	3
**9**	76	Male	24.16	Right kidney	4.5×3.3×1.5	Clear cell RCC	T3N0M0	3
**10**	72	Male	30.12	Right kidney	7.0×6.0×4.5	Clear cell RCC	T1bN2M0	3
**11**	52	Male	25.78	Right kidney	3.8×3.5×3.5	Clear cell RCC	T1aN0M0	3
**12**	61	Female	23.83	Left kidney	6.0×4.5×4.0	Clear cell RCC	T1bN0M0	3

Supplementary Table 1, Clinic pathologic information of RCC patients whose samples were analyzed in this study are listed in the table.

Sample ID	Age (years)	Gender	BMI (Kg/m^2)	Tumor site	Tumor size (cm)	Pathology	BCLC stage
**1**	63	Male	27.22	Left lobe	4.1×3.5×2.8	HCC	B
**2**	52	Male	23.88	Right lobe	2.6×2×1	HCC	A1
**3**	51	Male	22.59	Right lobe	4.0×3.6×2.6	HCC	A1
**4**	53	Female	25.83	Right lobe	8.0×7.0×6.0	HCC	A2
**5**	63	Male	23.88	Right lobe	5.0×4.5×3.5	HCC	A2
**6**	71	Male	21.22	Left lobe	6.7×5.5×4.2	HCC	A3
**7**	36	Male	20.76	Right lobe	2.7×2.2×2.2	HCC	C
**8**	55	Female	36.68	Right lobe	1.5×1.5×1.5	HCC	A1
**9**	50	Male	19.37	Left lobe	4.3×3.2×2.2	HCC	A3
**10**	43	Male	27.04	Right lobe	2.0×2.0×1.5	HCC	A1
**11**	52	Male	25.16	Left lobe	10.0×8.0×6.0	HCC	B
**12**	60	Male	22.76	Right lobe	1.5×1.0×1.3	HCC	A1

Supplementary Table 2, Clinic pathologic information of HCC patients whose samples were analyzed in this study are listed in the table.

Sample ID	Age (years)	Gender	BMI (Kg/m^2)	Tumor site	Tumor size (cm)	Pathology	TNM stage	Grade
**1**	82	Male	25.41	Sigmoid colon	4.5×3.5×0.8	Adenocarcinoma	T3N1M0	III
**2**	62	Male	22.26	Right half colon	4.5×3.0×2.5	Adenocarcinoma	T2N0M0	II
**3**	63	Female	28.52	Left half colon	9.0×6.5×3.0	Adenocarcinoma	T3N2M0	III
**4**	81	Male	19.68	Right half colon	6.3×3.0×3.0	Adenocarcinoma	T3N1M0	III
**5**	44	Female	23.73	Left half colon	7.0×5.2×1.2	Adenocarcinoma	T3N0M0	II
**6**	59	Female	24.67	Sigmoid colon	3.0×2.0×0.5	Adenocarcinoma	T2N0M0	II
**7**	65	Male	18.02	Right half colon	5.0×4.5×1.5	Adenocarcinoma	T3N0M0	III
**8**	79	Female	26.78	Right half colon	8.0×4.5×1.5	Adenocarcinoma	T3N1M0	III
**9**	50	Male	22.45	Sigmoid colon	2.5×2.0×1.0	Adenocarcinoma	T2N0M0	I
**10**	63	Male	24.91	Left half colon	3.5×2.0×1.5	Adenocarcinoma	T2N0M0	II
**11**	67	Female	17.27	Transverse colon	4.5×4.0×0.6	Adenocarcinoma	T3N0M0	II
**12**	34	Male	23.15	Left half colon	5.0×3.5×2.0	Adenocarcinoma	T2N0M0	II

Supplementary Table 3, Clinic pathologic information of colon cancer patients whose samples were analyzed in this study are listed in the table.
